# Trajectory, healthcare utilisation and recovery in 3590 individuals with long covid: a 4-year prospective cohort analysis

**DOI:** 10.1136/bmjopen-2025-103884

**Published:** 2026-01-14

**Authors:** Jai Prashar, Toby Hillman, Emma C Wall, Amanpreet Sarna, Emma Mi, Robert Bell, Jagdeep Sahota, Michael Zandi, Patricia McNamara, Rebecca Livingston, Rebecca Gore, Catherine Lunken, Elena Bax, Rachel Nyam, Amir Masood Rafie Manzelat, Lyth Hishmeh, Emily Attree, Stephen Cone, Amitava Banerjee, Melissa Heightman

**Affiliations:** 1 University College London Hospitals NHS Foundation Trust, London, England, UK; 2 The Royal London Hospital, Barts Health NHS Trust, London, England, UK; 3 The Francis Crick Institute, London, UK; 4 Centre for Clinical Pharmacology and Precision Medicine, Queen Mary University of London, London, England, UK; 5 Nuffield Department of Primary Care Health Science, University of Oxford, Oxford, England, UK; 6 Department of Neuroinflammation, UCL Queen Square Institute of Neurology, London, England, UK; 7 Long Covid SOS, London, UK; 8 Ridgmount Practice, London, UK; 9 Institute of Health Informatics, University College London, London, UK; 10 Respiratory Medicine, University College London Hospitals NHS Foundation Trust, London, UK

**Keywords:** COVID-19, Epidemiology, Health Services, Health policy, INTERNAL MEDICINE

## Abstract

**ABSTRACT:**

**Objective:**

To characterise long-term trajectory of recovery in individuals with long covid.

**Design:**

Prospective cohort.

**Setting:**

Single-centre, specialist post-COVID service (London, UK).

**Participants:**

Individuals aged ≥18 years with long covid (hospitalised and non-hospitalised) from April 2020 to March 2024.

**Main outcome measures:**

Routine, prospectively collected data on symptoms, quality of life (including Fatigue Assessment Scale (FAS) and EuroQol 5 Dimensions (EQ-5D), return to work status and healthcare utilisation (investigations, outpatient and emergency attendances). The primary outcome was recovery by self-reported >75% of ‘best health’ (EQ-5D Visual Analogue Scale) and was assessed using Cox proportional hazards regression models over 4 years. Linked National Health Service England registry data provided secondary care healthcare utilisation and expenditure.

**Results:**

We included 3590 individuals (63.3% female, 73.5% non-hospitalised, median age 50.0 years, 71.9% with ≥2 doses of COVID-19 vaccination), who were followed up for a median of 136 (0–346) days since first assessment and 502 (251–825) days since symptom onset. At first assessment, 33.2% of employed individuals were unable to work. Dominant symptoms were fatigue (78.7%), breathlessness (68.1%) and brain fog (53.5%). 33.4% of individuals recovered to >75% of best health prior to clinic discharge (recovery occurred median 202 (94–468) days from symptom onset). Vaccinated individuals were more likely to recover faster (pre: HR 2.93 (2.00–4.28) and post: HR 1.34 (1.05–1.71) COVID-19 infection), whereas recovery hazard was inversely associated with FAS (HR 0.37 (0.33–0.42)), myalgia (HR 0.59 (0.45–0.76)) and dysautonomic symptoms (HR 0.46 (0.34–0.62)). There was high secondary care healthcare utilisation (both emergency and outpatient care). Annual inpatient and outpatient expenditure was significantly lower in hospitalised individuals while under the service. When compared with the prereferral period, emergency department attendances were reduced in non-hospitalised patients with long covid, but outpatient costs increased.

**Conclusions:**

In the largest long covid cohort from a single specialist post-COVID service to date, only one-third of individuals under follow-up achieved satisfactory recovery. Fatigue severity and COVID-19 vaccination at presentation, even after initial COVID-19 infection, was associated with long covid recovery. Ongoing service provision for this and other post-viral conditions is necessary to support care, progress treatment options and provide capacity for future pandemic preparedness. Research and clinical services should emphasise these factors as the strongest predictors of non-recovery.

**STRENGTHS AND LIMITATIONS OF THIS STUDY**

- This study investigates long-term follow-up of long covid in the largest specialist post-COVID service in the UK.
- Diagnosis of long covid is adjudicated and validated by specialist clinicians.
- Use of routine electronic health record data increases the clinical applicability of findings.
- Validated tools (eg, EuroQol 5 Dimensions and Fatigue Assessment Scale for symptom burden) were used, enabling comparability with other long covid research.
- This single-centre analysis is a cohort with severe long covid, requiring specialist care, which may affect generalisability of findings.
- Trajectory, healthcare utilisation and recovery in 3590 individuals with long covid: a prospective cohort analysis.

## INTRODUCTION

 Post-COVID syndrome, or long covid (LC), continues to significantly impact both individuals and health systems,^1 2^ with over 2 million people estimated to be affected in the UK^3^ and major long-term impacts on healthcare utilisation.^4 5^ Definitions of LC are broad, diagnostic tests are still lacking, disease trajectory is poorly defined, few clinical trials are underway and there is currently no high-quality evidence base for therapeutic effectiveness.^6^ Commissioning guidance for post-COVID services was updated in December 2023 with the intention of ongoing provision of care following this model. However, funding instability is increasingly leading to significant variations in design and delivery of these specialist services across the UK,^7^ which is of serious concern.

Research into healthcare utilisation by specialist LC services to date has been limited by relatively small, selected observational studies, electronic health record (EHR) analyses using varying LC definitions and focus on post-hospitalised (PH) populations, frequently omitting the majority of individuals with LC who were non-hospitalised (NH).^8^ The US Veterans’ Affairs database has provided crucial insights into post-COVID-19 prognosis,^9^ but LC coding is heterogeneous, such study populations are predominantly male and white, and patient-reported outcome measures (PROMs) are frequently missing, meaning the findings may not be generalisable to the wider LC population. Furthermore, it has been difficult to date to evaluate particular care models due to patient and clinical service heterogeneity.^10 11^

Reports of long-term follow-up throughout the pandemic, across different waves, with detailed patient-level recovery data, both clinician-reported and patient-reported, are scarce.^12^ To address this knowledge gap, we analysed longitudinal routine EHR data (2020–2024) from the University College London Hospitals (UCLH) National Health Service (NHS) Trust, a specialist, multiprofessional LC service opened in April 2020^13^ (online supplemental figure S1), that developed a clinical model followed by 100 LC clinics in England and other countries.^14^ We report the trajectory of LC and utility of specialist LC services, including characteristics at first assessment, symptom burden, factors associated with functional recovery and healthcare utilisation in our unique patient cohort.

## METHODS

### Study design

We conducted a prospective, longitudinal cohort study, including electronic patient questionnaires and routine EHR data (UCLH LC service), and healthcare utilisation (NHS England LC registry). Patients were seen in the UCLH LC service in an initial assessment, with follow-up appointments arranged based on clinical judgement.

### Study population

Two cohorts were included in this study: first,we included individuals aged ≥18 years with LC (both PH and NH) attending the UCLH LC service presenting with suspected long covid from 1 April 2020 (clinical inception) until 31 March 2024*,* with valid referral source, self-rated health, vaccination status, comorbidities and symptoms. As previously published, the clinic accepts referrals from three sources: (1) PH: postadmission to UCLH with COVID-19; (2) NH: individuals referred from primary care with suspected long covid ≥6 weeks post-SARS-CoV-2 infection; and (3) post emergency department: referral for individuals with persistent symptoms at 4–6 weeks after attendance.^13^ We included patients with both a valid completed Fatigue Assessment Scale (FAS) score and date of symptom onset in the regression analyses.

Second, for patients presenting between 11 May 2020 and 30 November 2023, we included a LC registry-based cohort from NHS England. Data was available for these patients from 1 January 2018 to end of follow-up, defined as earliest of date of death (if applicable) or 28 February 2024 (for admitted patient care and emergency care analysis) or 31 March 2023 (for outpatient (OP) analysis).

### Setting

The UCLH LC service started telephone consults for PH patients in April 2020 and opened to in-person attendances on 11 May 2020, receiving referrals from primary and secondary care. The multidisciplinary clinic includes physicians (respiratory, cardiology, neurology and infectious diseases), physiotherapists, occupational therapists, psychologists, dedicated administrator and nurse coordinator support, and is fully described in online supplemental methods.

### Data sources

From May 2020, structured clinical assessments were implemented using the Epic^TM^ EHR. A patient-facing electronic questionnaire documenting symptoms, PROMs such as the FAS,^15^ EuroQol 5 Dimensions (EQ-5D) Visual Analogue Scale (VAS)^16^ and impact on work and function was implemented in December 2020 and completed at first and follow-up assessments. Triggering SARS-CoV-2 variant was deduced from estimated time point of infection causing ongoing illness, referenced against UK Health Security Agency data on circulating variants. Patient-level data on LC service activity was submitted to the national LC Registry, enabling NHS England-led analysis of wider OP and other healthcare utilisation. Did-not-attend rates were low in initial analyses (5–6%) and were not analysed further as part of this work.

For healthcare utilisation, follow-up was divided into three periods:

- ‘Pre-COVID’: Before COVID-19 pandemic (1 January 2018 to 31 December 2019).
- ‘Pre-assessment’: From LC onset (4 weeks after first positive SARS-CoV-2 test/onset of COVID-19 symptoms for those unable to test) to initial assessment in the LC service.
- ‘In-service’: From initial assessment in the LC service to end of follow-up.

Further details are in online supplemental methods.

Variables: Available variables at each assessment included FAS (for which a higher score was associated with more severe fatigue), return-to-work status, symptoms and comorbidities (table 1). These measurements were collected at initial and subsequent assessments based on clinical need.

**Table 1:** Characteristics at first assessment and follow-up in 3590 individuals with long covid

	Overall (n=3590)	PH (n=953)	NH (n=2637)	P value
Median age (years (IQR)	50.0 (39.0–60.0)	59.0 (48.0–69.0)	47.0 (37.0–56.0)	<0.001
Female gender	2273 (63.3%)	429 (45.0%)	1844 (70.0%)	<0.001
Ethnicity				
Black	240 (6.7%)	125 (13.1%)	115 (4.4%)	<0.001
White	1626 (45.3%)	386 (40.5%)	1240 (47.0%)	0.010
Asian	306 (8.5%)	119 (12.5%)	187 (7.1%)	<0.001
Other	233 (6.5%)	87 (9.1%)	146 (5.5%)	<0.001
Mixed	98 (2.7%)	23 (2.4%)	75 (2.8%)	0.490
Unknown	1025 (28.6%)	212 (22.3%)	813 (30.8%)	<0.001
COVID-19 vaccination status				
Full	2581 (71.9%)	724 (76.0%)	1857 (70.4%)	0.08
Full pre-COVID-19 infection	330 (9.2%)	49 (5.1%)	281 (10.7%)	<0.001
Employment status				
Usually employed and able to work	1789 (49.8%)	300 (31.5%)	1489 (56.5%)	<0.001
Usually employed and unable to work	891 (24.8%)	217 (22.8%)	674 (25.6%)	0.14
Not employed	250 (7.0%)	85 (8.9%)	165 (6.3%)	0.01
Retired	280 (7.8%)	161 (16.9%)	119 (4.5%)	<0.001
Unknown	380 (10.6%)	190 (19.9%)	190 (7.2%)	<0.001
Characteristics at first assessment				
Number of symptoms (median (IQR)	5.0 (3.0–9.0)	5.0 (2.0–16.0)	5.0 (3.0–9.0)	0.13
% of best health, Visual Analogue Scale	50.0 (30.0–70.0)	65.0 (40.0–85.0)	50.0 (30.0–66.0)	<0.001
EQ-5D score, overall (median (IQR)	45 (29.0–61.0)	49.0 (25.0–70.0)	45.0 (30.0–60.0)	0.26
Recovered at first assessment*	805 (22.4%)	383 (40.2%)	422 (16.0%)	<0.001
Days since symptom onset (median (IQR)†	236 (119.0–450.0)	117.0 (84.0–269.25)	282.0 (151.0–496.0)	<0.001
Variant triggering infection				
Wild-type	1459 (40.6%)	325 (34.1%)	1134 (43.0%)	<0.001
Alpha	1236 (34.4%)	463 (48.6%)	773 (29.3%)	<0.001
Delta	423 (11.8%)	136 (14.3%)	287 (10.9%)	<0.001
Omicron	472 (13.2%)	29 (3.0%)	443 (16.8%)	0.01
Comorbidities				
Median (IQR) number of comorbidities per group, n	1 (1–2)	2 (1–3)	1 (0–1)	<0.001
Asthma	560 (15.6%)	130 (13.6%)	430 (16.3%)	0.07
Hypertension	553 (15.4%)	309 (32.4%)	244 (9.3%)	<0.001
Diabetes mellitus, type 2	288 (8.0%)	188 (19.7%)	100 (3.8%)	<0.001
Migraine	262 (7.3%)	27 (2.8%)	235 (8.9%)	<0.001
Depression	247 (6.9%)	50 (5.3%)	197 (7.5%)	0.025
Hypercholesterolaemia	215 (6.0%)	114 (12.0%)	101 (3.8%)	<0.001
Hyperthyroidism	222 (6.2%)	64 (6.7%)	158 (6.0%)	0.44
GORD	179 (5.0%)	59 (6.2%)	120 (4.6%)	0.052
Anxiety	131 (3.7%)	16 (1.7%)	115 (4.4%)	<0.001
Irritable bowel syndrome	116 (3.2%)	13 (1.4%)	103 (3.9%)	<0.001
Osteoarthritis	103 (2.9%)	55 (5.8%)	48 (1.8%)	0.49
Fibromyalgia	111 (3.1%)	14 (1.5%)	97 (3.7%)	<0.001
Chronic obstructive pulmonary disease	70 (2.0%)	36 (3.8%)	34 (1.3%)	<0.001
Interstitial lung disease	77 (2.1%)	62 (6.5%)	15 (0.6%)	<0.001
Chronic fatigue syndrome	86 (2.4%)	4 (0.4%)	82 (3.1%)	<0.001
Ischaemic heart disease	69 (1.9%)	44 (4.6%)	25 (1.0%)	0.022
Polycystic ovarian syndrome	68 (1.9%)	11 (1.2%)	57 (2.2%)	0.053
Iron deficiency anaemia	46 (1.3%)	18 (1.9%)	28 (1.1%)	0.053
Diabetes mellitus, type 1	20 (0.6%)	6 (0.6%)	14 (0.5%)	0.72
Symptoms at first assessment				
Fatigue	2826 (78.7%)	696 (73.0%)	2130 (80.8%)	0.021
Cough	1208 (33.7%)	460 (48.3%)	748 (28.4%)	<0.001
Breathlessness	2443 (68.1%)	706 (74.1%)	1737 (65.9%)	0.010
Chest pain	1329 (37.0%)	442 (46.4%)	887 (33.6%)	<0.001
Chest tightness	1483 (41.3%)	429 (45.0%)	1054 (40.0%)	0.038
Vertigo	759 (21.1%)	323 (33.9%)	436 (16.5%)	<0.001
Brain fog or confusion	1922 (53.5%)	470 (49.3%)	1452 (55.1%)	0.037
Arthralgia	1442 (40.2%)	456 (47.9%)	986 (37.4%)	<0.001
Disturbed sleep	1637 (45.6%)	462 (48.5%)	1175 (44.6%)	0.13
Focal weakness	1222 (34.0%)	405 (42.5%)	817 (31.0%)	<0.001
Skin rash	737 (20.5%)	323 (33.9%)	414 (15.7%)	<0.001
Postural symptoms	1427 (39.8%)	411 (43.1%)	1016 (38.5%)	0.05
Myalgia	1666 (46.4%)	463 (48.6%)	1203 (45.6%)	0.25
No symptoms	442 (12.3%)	281 (29.5%)	161 (6.1%)	<0.001
Follow-up†				
Median (IQR) number of assessments	2 (1.0–4.0)	2 (1.0–3.0)	2 (1.0–4.0)	<0.001
Median (IQR) duration from initial assessment to discharge (days)	136 (0–346)	77 (0–289.4)	152 (0–364)	<0.001
Recovery during study period (n (%)	1198 (33.4%)	448 (47.0%)	750 (28.4%)	<0.001
Symptom onset to functional recovery (median duration (IQR), days)	202 (94–468)	108 (84–187)	316 (141–584)	<0.001
Median (IQR) number of follow-up assessments to functional recovery	1 (1.0–2.0)	1 (1.0–2.0)	1 (1.0–2.0)	<0.001
Symptom onset to discharge (median duration (IQR), days)	502 (251–825)	333 (124–676)	542 (310–893)	<0.001

P value (comparing PH and NH individuals) is from χ2 test for categorical variables, and Mann-Whitney U test for continuous variables.

* >75% health

† n= 3497. 93 patients did not have a valid initial PROM date and were excluded from the regression analysis.

EQ-5D, EuroQol 5 Dimensions; GORD, gastro-oesophageal reflux disease; NH, non-hospitalised; PH, post-hospitalised; PROM, patient-reported outcome measure.

For the Cox proportional hazards regression model, the primary endpoint was self-reported ‘75% of best health’ in answer to the EQ-5D VAS (‘What percentage of your best health do you feel?’) as an established threshold used by UK LC services, supported by qualitative data.^17 18^ This endpoint was routinely measured at the initial and subsequent assessments.

Variable selection for these models involved L1/L2 regularisation and addition of other clinically important variables, as described in the online supplemental material. For the healthcare utilisation analysis, outcomes were annual rate of emergency admission inpatient (IP) days and care cost, emergency department (ED) attendances and care cost and OP attendances and care cost. OP attendances included clinical specialities related to LC (excluding LC service). As only limited sociodemographic explanatory variables were available for analysis, variables were selected based on clinical and investigator judgement.

### Statistical methods

#### Characteristics at first assessment

Descriptive characteristics are presented as percentages for categorical variables, mean (SD) for normally distributed continuous variables and median (IQR) for non-normally distributed continuous variables.

### Symptom burden

We used the Wilcoxon signed-rank test to determine significance of differences between first and last PROM in followed-up individuals. For characteristics at first assessment and outcomes, Mann-Whitney U test was used for continuous variables and χ^2^ test (with expected estimates adjusted for baseline prevalence) for categorical variables. For missing data for recorded date of follow-up PROMs (but not other variables such as functional recovery and healthcare utilisation), multivariate imputation was performed using a random forest estimator with recovered status, age, sex, FAS score and pre-COVID vaccination status as covariates in Python. Making the assumption that any missing data was missing at random, we chose random forest estimation for its relative robustness to non-normality and mixed data types. Imputation was only applied to individuals with partial missing data remaining under follow-up.

### Functional recovery

Cohen’s *d* was used to indicate effect size for changes in FAS, EQ-5D score and EQ-5D VAS.

We used Cox proportional hazards models, stratified by hospitalisation status and age group (18–44, 45–64 and 65+) to investigate the relationship between patient characteristics and self-reported recovery. Further details, including regarding variable selection, are in online supplemental methods. Two-tailed p<0.05 was considered statistically significant. Python V.3.10.8 was used for analyses.

#### Healthcare utilisation

Cost for OP care was calculated by multiplying each activity by relevant unit cost. To account for changes in background rates over time, healthcare utilisation rates in pre-assessment and in-service periods were standardised to the pre-COVID period.

Changes in healthcare utilisation (IP, ED and OP) between follow-up periods were studied by Wilcoxon signed-rank test and multiple linear regression for associated factors: demographic (age at in-service assessment, gender, ethnicity, socioeconomic deprivation), service-related (duration of follow-up, time until assessment) and vaccination status. PH and NH subgroups were analysed. Two-tailed p<0.05 was statistically significant. R V.3.8 was used for healthcare utilisation analyses.

#### Data availability

Anonymised study data can be made available on reasonable request, with completion of appropriate data sharing agreements and approval by relevant data access committees.

## RESULTS

### Characteristics at first assessment

**Figure 1:**
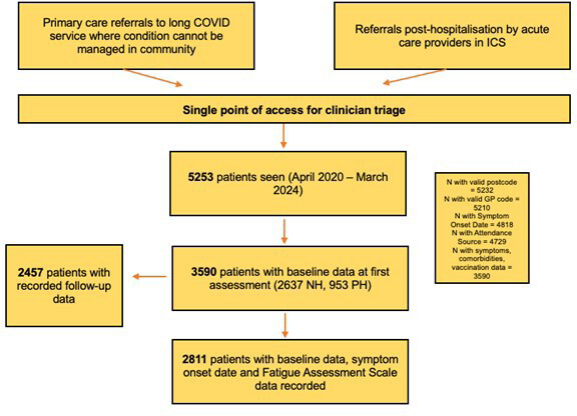
The integrated post-COVID pathway in the University College London Hospitals NHS Trust long covid service, study population and follow-up. GP, general practitioner; ICS, Integrated Care System; NH, non-hospitalised; NHS, National Health Service; PH, post-hospitalised.

3590 (26.5% PH and 73.5% NH) individuals were included (figure 1). 63.3% were female (45.0% in PH). The median age was 50.0 years (PH 59.0 vs NH 47.0). 2457 individuals (68.4%) were followed up (PH 59.3% and NH 71.7%) (table 1). Median follow-up was 136 (0–346) days, and median time from symptom onset to discharge was 502 (251–825) days.

Overall, white ethnicity was most common (45.3%), but black and Asian ethnicity were more common among PH than NH individuals (13.1% and 12.5% vs 4.4% and 7.1%, p<0.001). Most (PH 82.7% and NH 72.3%) individuals reported symptom onset early in the pandemic (2020–2021). In the Omicron era, 93.9% of 472 cases were NH (table 1). 71.9% were fully COVID-19 vaccinated (had received two or more vaccine doses) at first assessment. Asthma (15.6%), hypertension (15.4%) and type 2 diabetes mellitus (8.0%) were common pre-existing diagnoses. There were no significant differences compared between those with baseline characteristics and with the cohort with sufficient data available (see Methods) included in Cox regression models (n=2811) (online supplemental table S1). The registry cohort included 4578 individuals (PH 25.5%): median age 49.0 years, 61.7% female and 28.9% fully COVID-19 vaccinated pre-acute COVID-19 (online supplemental table S2).

### Symptom burden

Initial symptoms (median 5 (3–9)) were commonly fatigue (78.7%), dyspnoea (68.1%), brain fog (53.5%), myalgia (46.4%) and disturbed sleep (45.6%) (table 1). Initial symptom frequency was similar across variants (online supplemental figure S2 and S3). 77.6% reported <75% of ‘best health’ (mean ‘best health’: PH 65.0% vs NH 50.0%). 89.4% of individuals provided employment status, of whom, 74.7% were employed (33.2% of whom reported being unable to work), 7.8% retired and 7.0% unemployed.

### Functional recovery

**Figure 2:**
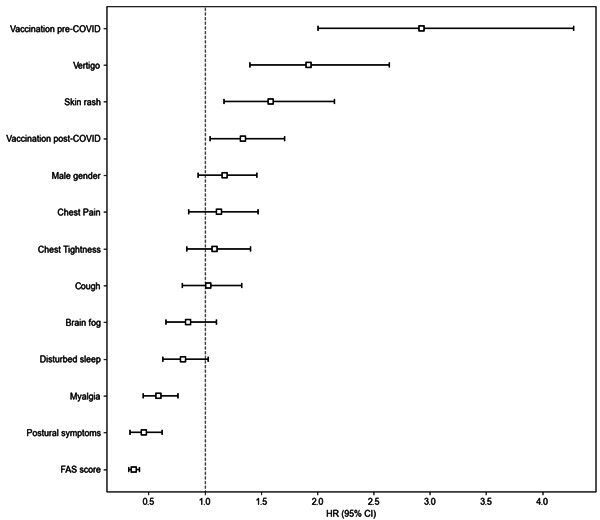
Factors associated with recovery at first assessment in individuals with long covid*. *Cox proportional hazards regression model showing factors associated with spontaneous recovery at first assessment (n=2811). Recovery is defined as subjective attainment of >75% of best health by first assessment. FAS, Fatigue Assessment Scale.

**Figure 3:**
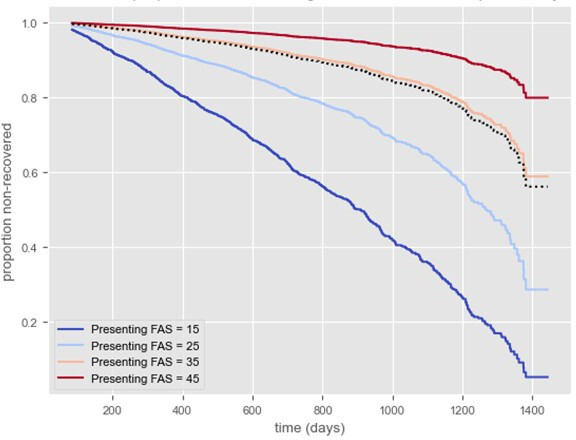
Recovery over 4 years in individuals with long covid, stratified by fatigue severity on initial assessment. n=2198 individuals with a valid symptom onset date and FAS score at follow-up. Black dotted line=mean presenting FAS. FAS, Fatigue Assessment Scale.

The proportion with complete follow-up and functional recovery data at first and last assessment is shown in online supplemental file 1. As fatigue was the most frequently reported symptom, we compared FAS between first and last assessment in the service, across both PH and NH patients (n=2266). Overall, median reported FAS was 33 (25–39) at first appointment, compared with 31 (24–39) at the last assessment. Median FAS was higher in the NH compared with the PH cohorts (table 2). Similarly, there was minimal change in median EQ-5D (n=2085; 45 (30–60) and 50 (30–65), Cohen’s *d=*0.188) and increased median best health (n=2456; 50.0% (30.0%–65.0%) to 57.0% (36.0%–75.0%), Cohen’s *d*=0.219) from first to last assessment in followed-up individuals (table 2). 1198 (33.4%) of individuals recovered to 75% of best health during follow-up (NH 28.4% and PH 47.0%; p<0.001). Median symptom onset to recovery (defined as self-reported ≥75% of best health) was 202 (94–468) days: higher in NH than PH (p<0.001)(table 3). COVID-19 vaccination, whether pre (HR 2.93 (2.00–4.28)) or post (1.34 (1.05–1.71)) COVID-19 infection, vertigo (1.92 (1.40–2.64)) and skin rash (1.59 (1.17–2.15)) were associated with recovery (online supplemental table S4). FAS (0.37 (0.33–0.42)), myalgia (0.59 (0.45–0.76)) and postural symptoms/dysautonomia (0.46 (0.34–0.62)) at first assessment were inversely associated with recovery (figure 2). Associations were similar in PH and NH groups (online supplemental S4 figures and S5)**,** although male gender had a significant protective effect in the NH group. Patients reporting FAS >45 were associated with the lowest recovery rates after stratification by fatigue severity (figure 3).

**Table 2:** Completeness of and changes in PROM scores between first and last assessment in followed-up individuals with long covid

	NH (n=2637)			PH (n=953)			Overall (n=3590)		
	First	Last	P value (Cohen’s *d*)	First	Last	P value (Cohen’s *d*)	First	Last	P value (Cohen’s *d*)
FAS									
Completeness	2274 (86.2%)	1829 (69.4%)		543 (57.0%)	437 (45.9%)		2817 (78.5%)	2266 (63.1%)	
Score	33.0 (26.0–39.0)	32.0 (25.0–39.0)	<0.001 (−-0.106)	31.0 (22.0–38.0)	30.0 (23.0–38.0)	0.15 (*−*0.047)	33.0 (25.0–39.0)	31.0 (24.0–39.0)	<0.001 (−0.095)
EQ-5D (overall)									
Completeness	2181 (82.7%)	1715 (65.0%)		493 (51.7%)	370 (38.8%)		2674 (74.5%)	2085 (58.1%)	
Score	45.0 (30.0–60.0)	50.0 (30.0–65.0)	<0.001 (0.189)	48.0 (25.0–66.0)	50.0 (30.0–70.0)	<0.001 (0.180)	45.0 (29.0–60.0)	50.0 (30.0–66.0)	<0.001 (0.188)
Percentage of best health, VAS (%)									
Completeness	2637 (100.0)	1877 (71.2)		953 (100.0)	559 (58.7)		3590 (100.0)	2436 (67.9)	
Score	45.0 (30.0–60.0)	51.0 (35.0–70.0)	<0.001 (0.217)	59.0 (34.0–80.0)	66.0 (40.0–85.0)	<0.001 (0.228)	50.0 (30.0–65.0)	57.0 (36.0–75.0)	<0.001 (0.219)

P value is from Wilcoxon signed-rank test.

EQ-5D, EuroQol 5 Dimensions; FAS, Fatigue Assessment Scale; NH, non-hospitalised; PH, post-hospitalised; PROMs, patient-reported outcome measures; VAS, Visual Analogue Scale.

**Table 3:** Healthcare utilisation and costs per year during pre-COVID, pre-assessment and in-service periods for individuals with long covid

	Pre-COVID, median (IQR)	Pre-assessment, median (IQR)	In-service, median (IQR)	Change from pre-assessment to in-service		Change from pre-COVID to pre-assessment	
				Pseudomedian (95% CI)	P value	Pseudomedian (95% CI)	P value
Overall							
IP days	0 (0–0)	0 (0–0)	0 (0–0)	−0.06 (−0.54 to 0.40)	0.787	1.59 (1.01 to 2.29)	<0.001
IP cost, £	0 (0–0)	0 (0–0)	0 (0–0)	15 (−208 to 237)	0.897	368 (135 to 616)	0.001
ED attendances	0 (0–0.57)	0.26 (0–1.48)	0 (0–0.82)	−0.44 (−0.51 to −0.38)	<0.001	0.79 (0.72 to 0.87)	<0.001
ED cost, £	0 (0–92)	27 (0–212)	0 (0–121)	−64 (−76 to −54)	<0.001	128 (115 to 141)	<0.001
OP appts	0.50 (0–2.00)	1.03 (0–5.62)	2.71 (0.77–6.44)	0.95 (0.78 to 1.13)	<0.001	2.24 (2.00 to 2.49)	<0.001
OP cost, £	89 (0–401)	202 (0–1092)	523 (150–1247)	187 (152–221)	<0.001	455 (409–503)	<0.001
Hospitalised							
IP days	0 (0–0)	0.57 (0–3.14)	0 (0–0.35))	−1.21 (−2.84 to 0.03)	0.055	6.40 (4.26 to 9.07)	<0.001
IP cost, £	0 (0–0)	482.24 (0–1127)	0 (0–562))	−675 (−1416 to −71)	0.028	2326 (1393 to 3484)	<0.001
ED attendances	0 (0–0.57)	0 (0–1.15)	0.38 (0–1.07)	−0.02 (−0.18 to 0.18)	0.8	0.77 (0.54 to 0.99)	<0.001
ED cost, £	0 (0–106.72)	0 (0–163)	60 (0–184)	19 (−13 to 44)	0.252	122.89 (82 to 169)	<0.001
OP appts,	0.5 (0–3)	2.90 (0.57–10.56)	2.66 (0.53–6.49)	−0.94 (−1.62 to −0.32)	0.004	5.27 (4.40 to 6.18)	<0.001
OP cost, £	98 (0–531)	604 (170–2054)	510 (110–1226)	−195 (−322 to −75)	0.002	1054 (881 to 1231)	<0.001
Non-hospitalised							
IP days	0 (0–0)	0 (0–0)	0 (0–0)	0.35 (−0.03 to 0.71)	0.075	0.08 (−0.24 to 0.48)	0.555
IP cost, £	0 (0–0)	0 (0–0)	0 (0–0)	244 (46 to 432)	0.015	−149 (−331 to 23)	0.092
ED attendances	0 (0–0.57)	0.31 (0–1.55)	0.24 (0–0.76)	−0.58 (−0.65 to −0.51)	<0.001	0.81 (0.73 to 0.89)	<0.001
ED cost, £	0 (0–79)	21.71 (0–220)	0 (0–104)	−88 (−100 to −77)	<0.001	131 (118 to 145)	<0.001
OP appts	0.5 (0–2)	1.11 (0–4.4)	2.73 (0.81–6.41)	1.40 (1.21 to 1.59)	<0.001	1.57 (1.37 to 1.78)	<0.001
OP cost, £	86.56 (0–346)	214.24 (0–868)	532 (163–1260)	278 (241 to 316)	<0.001	324 (284 to 365)	<0.001

Change between periods determined by Wilcoxon signed-rank test.

appts, appointments; ED, emergency department; IP, inpatient; OP, outpatient.

### Healthcare utilisation (registry-linked analyses)

Overall, IP days, ED and OP attendances and cost increased significantly between the pre-COVID to LC periods (table 2). Following access to LC service, we found reduced ED attendances per year (−0.44 days, p<0.001), saving £64 (£54 to £76) per person, and increased OP attendances (0.95, p<0.001) with increased cost of £187 (£152 to £221, p*<*0.001)(table 2). For PH individuals, there was no reduction in IP days after LC service access (−1.21 days (0.03 to −2.84 days, p*=*0.055)), however, there was a reduced annual IP cost (−£675 (−£71 to −£1416, p=0.028)), one less OP attendance per year (−0.94 days (−0.32 to −1.62, p=0.004)) and reduced OP cost (£195 (£75 to £322, p=0.002)). NH individuals had more OP attendances, fewer ED attendances per year and reduced cost. Following LC service access, total healthcare-associated cost was higher for ED attendances in older individuals, OP in females and IP days in least deprived individuals. Black ethnicity was associated with reduced OP costs. Increased time from LC onset to LC service assessment was associated with reduced OP savings (online supplemental table S5).

## DISCUSSION

In the largest analysis of a dedicated specialist LC service to date, we present three findings. First, NH individuals represent three-quarters of LC service users with comorbidities, symptoms, recovery and healthcare utilisation comparable to PH individuals. Second, despite LC symptom heterogeneity, fatigue correlates negatively with long-term recovery. Third, short- and long-term functional recovery is very low over the median follow-up period of 502 days (for non-hospitalised individuals: 16% spontaneous recovery at initial assessment and 28% during the follow-up period). Our analysis of EQ-5D VAS severity of long covid is comparable to scores seen in myalgic encephalomyelitis/chronic fatigue syndrome^19^ and worse than in some cohorts with chronic conditions such as heart failure and chronic obstructive pulmonary disease.^20 21^

LC can cause morbidity at least 3 years after onset, but to date, the focus has been on PH individuals, where ‘risk of death declined but remained significantly elevated in the third year after infection’.^22^ We now add that most individuals presenting to LC services are NH with significant healthcare needs, approachable/manageable using a specialist clinical pathway. Preparedness and response across countries has, both historically and during the COVID-19 pandemic, mainly focused on acute, hospitalised individuals with some exceptions, usually population-wide EHR studies.^23 24^ Recent cross-country experience of a ‘quad-demic’ of influenza, COVID-19, norovirus and respiratory syncytial virus,^25 26^ with increasing acute infections, should signal the threat of chronic effects of high prevalence of infections. Our results suggest that LC, particularly NH individuals, necessitates ongoing care. This analysis, combined with evidence from the management of the pandemic and delivery of post-COVID care, supports the development of integrated care models to tackle future health shocks.^27^

Research to date highlights variation in LC symptoms, but efforts towards subtypes or biomarkers are yet to establish optimal risk predictors of recovery^28^ or standardised assessment tools,^29 30^ limiting clinical applicability.^31^,^34^ The most severely fatigued individuals, where disease mechanisms are unclear,^35^ are not prioritised in care and research. We show that fatigue and quality of life, simply assessed by FAS and EQ-5D, correlate well with prognosis and can be used in practice to identify those in most need of integrated care.

Recovery rates were low with significant healthcare utilisation, both within and outside the LC service. Over two-thirds of individuals required follow-up and approximately 20% had five visits prior to discharge. Consistent with other studies, we demonstrated substantially increased healthcare utilisation and cost post-LC onset, compared with prepandemic. LC service care was linked to reduction in wider healthcare utilisation for PH individuals and in ED use for NH individuals, suggesting value of LC services in reducing overall disease burden and healthcare. Early intervention may be associated with better outcomes by reducing fatigue and other symptoms, or by reducing the severity of the underlying disease process, but further studies with longer follow-up are required, including benchmarking of cost of LC services. COVID-19 vaccination before COVID-19 infection was associated with improved time to recovery by twofold on average, supporting the protective effect of vaccination in developing LC.^36 37^ This association underpins the importance of tackling vaccine hesitancy and low uptake, which is evident from our cohort (where only 9.2% of individuals were fully vaccinated pre-index COVID infection).

There are important clinical implications. First, LC is a multisystem disorder,^31^ which often requires multidisciplinary input for effective care. Second, fatigue must be assessed and severe fatigue prioritised, since it is a cardinal feature of functional limitation in LC,^38^ is highly prevalent and has key prognostic significance. Third, like other cohorts,^39^ we show prolonged recovery, highlighting the need for continuity of care in dedicated specialist services.

There are three main research and policy implications. First, we show the value of routine clinical EHR data and routinely collected PROMs, offering a ‘learning health system’ approach which should be prioritised more widely in clinical care,^27^ which has not been a focus for research funding for LC so far.^6^ Second, a multidisciplinary, integrated LC service, as established in several contexts,^40^,^42^ can reduce morbidity and long-term healthcare expenditure in NH and PH individuals, with likely cost-effectiveness. Third, our observed low vaccination rates suggest that COVID-19 vaccination should be emphasised, available and accessible, and its benefit is likely to be concentrated in the pre-assessment period and ideally pre-COVID-19 infection.

This work also suggests that fatigue—a symptom without known pathogenesis or established management in LC—is central to the extent and duration of morbidity in LC, supported by other work.^38^ There is some suggestion that rehabilitative interventions may offer benefit,^43^ although the direct impact of multidisciplinary care on long-term fatigue remains poorly elucidated.

Our use of validated tools (eg, EQ-5D and FAS for symptom burden) and standardised statistical analysis enables comparability with other research in LC and other chronic conditions. However, we note some limitations. In this single-centre analysis is a cohort with severe LC, requiring specialist care, which may affect generalisability of our findings—giving way to a possible ‘framing effect’^44^ where the relative prevalences of symptoms and functional impairment may not faithfully represent that in the general population. While there is a theoretical possibility that some participants may not have expressed the full range of symptoms at first assessment, the long median time from symptom onset to first assessment makes this unlikely. Further, we were unable to fully exclude biases towards those with greater illness severity, who were most likely to have longer follow-up and may have had more opportunities to provide responses, and bias against those achieving functional recovery before initial assessment may have chosen not to attend, or to self-discharge from follow-up. Additionally, as is common in clinical assessments, we were unable to compare outcomes with matched patients with other diseases, limiting our ability to fully determine the impact of LC clinic care. Finally, in this routine care dataset, there was considerable missingness in key parameters at follow-up, making it difficult to apply more sophisticated statistical techniques (although this is an interesting area for future analysis).

Despite these limitations, we report the largest real-world study of individuals in a specialist LC service to our knowledge, providing high-resolution detail, including comorbidities, symptomatology, functional impairment, employment status, trajectory of illness, healthcare utilisation and costs of LC to inform the ongoing care of this complex and vulnerable patient population.

## CONCLUSION

LC is associated with high healthcare utilisation and costs, with evidence of significant and prolonged functional impairment in this cohort, of whom the great majority are of working age. Certain symptoms may be key prognostic markers, including severity of fatigue, dysautonomia, myalgia and brain fog, with full COVID-19 vaccination associated with a protective effect. These findings justify the use of dedicated integrated services to meet these complex needs and may be more efficient for health systems, with NH and PH patients equally in need of specialist care. Maintaining both clinical provision and research into long COVID and other post-viral conditions will support future pandemic preparedness, enable rapid increase in capacity at times of future need and continue progress made to date to improve treatment options for those with ongoing significant illness due to COVID-19.

## Supplementary material

10.1136/bmjopen-2025-103884 online supplemental file 1

## Data Availability

No data are available.
