# Supplementary material for: Trajectory, healthcare utilisation and recovery in 3590 individuals with long covid: a 4-year prospective cohort analysis
Source: BMJ Open. 2026 Jan 14;16(1):e103884. doi: 10.1136/bmjopen-2025-103884 (PMC12815175; doi:10.1136/bmjopen-2025-103884)

Supplementary Material

*Trajectory, healthcare utilisation and recovery in 3590 individuals with long COVID: a 4-year prospective cohort analysis.*

**Contents**

**Supplementary Methods 2-4**

**Table S1.** Baseline characteristics of individuals with Long COVID in the UCLH LC service (regression cohort). **5-6**

**Table S2.** Baseline characteristics of Long COVID registry cohort at first Long COVID service assessment. **7**

**Table S3.** Follow-up and functional recovery in individuals with Long COVID with Patient-Reported Outcomes (PROMs) recorded at first and last assessment. **8**

**Table S4.** Results of Cox proportional hazards regression. **9**

**Table S5.** Factors associated with change in healthcare utilisation cost between Pre-assessment and Long COVID service periods in individuals with long COVID. **10**

**Figure S1.** Long COVID service structure at University College London Hospitals NHS Trust. **11**

**Figure S2.** COVID-19 variant and time to first presentation in individuals with Long COVID (*n*=2811). **12**

**Figure S3.** Proportion of individuals with Long COVID with symptoms at first presentation by triggering variant. **13**

**Figure S4.** Factors associated with recovery in non-hospitalised individuals with Long COVID in Cox proportional hazards regression

**14**

**Figure S5:** Factors associated with recovery in hospitalised individuals with Long COVID in Cox proportional hazards regression

**15**

**Supplementary Methods**

***Setting:*** The service was commissioned as the lead provider for the North Central London Integrated Care System (ICS). Post-COVID rehabilitation services were commissioned from April 2021 and were incorporated into an integrated pathway across the medically supported service and four community LC rehabilitation services. Training in the management of LC was made available to all required providers within the ICS. The service ran according to NHS England commissioning guidance from June 2021, which was updated in December 2023. A single point of access for referrals was established with triage enabled by a structured questionnaire **(Supplementary Figure 1)**. NH referrals were via general practice or community referral. After first assessment, where possible, patients were discharged with self-management advice according to symptom burden, or onward referral to post-COVID rehabilitation and/or psychological services. If further diagnostics or medical management were required, follow-up was arranged. Where investigations were completed, patients were discharged to patient-initiated follow up and could seek contact with the service if needed. Since April 2021, weekly activities include approximately 100 scheduled attendances**,** as well as multi-speciality, multi-disciplinary team (MDT) meetings, both hospital- and community-based (with primary care and post-COVID rehabilitation and psychology services) **(Supplementary Figure 2)**.

***Data:*** Due to lack of sequencing data from available diagnostic testing, further stratification by infecting variant was not possible. Within the registry, data were linked with other NHS England-held datasets at individual level, including demographic data, death data, Secondary Uses Service Admitted Patient Care (SUS APC), SUS OP and Emergency Care (SUS EC) data sets, COVID-19 test data from the Second Generation Surveillance System (SGSS), and COVID-19 vaccination data from the National Immunisation Management System (NIMS).

Where individuals lacked a recorded positive SARS-CoV-2 test in SGSS, onset of the COVID-19 pandemic, designated as 1st January 2020, was used as an estimate for COVID-19 acute infection. Full vaccination prior to first COVID-19 infection was defined as two doses of any COVID-19 vaccination at least 30 days before date of first SARS-CoV-2 positive test. Where individuals did not have a SARS-CoV-2 test record, the earliest of LC service referral date, triage date, or initial assessment date was used.

***Data analysis:***

Adherence to the proportional hazards assumption was tested using inspection of the Schoenfeld residuals and application of a rank transformation of the time scale, with variables demonstrating violations of the assumption included as a stratification variable. Continuous covariates were scaled to a Gaussian distribution with a mean of zero and unit variance. Given that there was significant variation in the service in time to first assessment, we opted to use time since the onset of index COVID infection (in days) for “time-to-event". For variable selection, L1/L2 regularization was first applied to all included coefficients, with L1 and L2 penalties applied to shrink the regression coefficients and select the most important features. The mixing parameter α was determined using 5-fold cross-validation, optimising for the concordance statistic. Additional clinically important characteristics were able to be added following this step if required to generate the final model; we added ‘cough’ and ‘skin rash’ as potential phenotypically important features within the main model. Models with the selected variables were fitted to the hospitalised and non-hospitalised cohorts.

A further model with the same variables (and presenting FAS score) was fitted on the whole cohort including repeat attendances, with a robust variance estimator used to cluster participants between attendances. Given the *a priori* expectation that presenting fatigue would exert a significant effect on recovery, survival curves were plotted while holding the FAS variable constant, in order to visualise the contributions of different levels of fatigue severity at presentation to long-term recovery.

Analysis of EHR data was performed via Microsoft SQL Server Management Studio and SQL extraction into an intermediate database, against which queries were run and statistical analysis performed using Python and associated libraries. Registry data analysis was performed in an NHS England secure data environment.

For the Cox proportional hazards model, using the techniques described in the *Methods*, we selected vaccination pre-COVID, vaccination post-COVID, age, male gender, total FAS score, cough, focal weakness, postural symptoms, myalgia, breathlessness, brain fog and disturbed sleep. Breathlessness was included as a stratification variable due to non-proportional hazards, along with hospitalisation status.

***Healthcare utilisation:*** Activity and cost of emergency admissions and ED attendances were derived from SUS APC and EC data of care episodes.

**Table S1. Baseline characteristics of individuals with Long COVID in the UCLH LC service (regression cohort).**

| **Variable** | **Overall (n=2811)** | **NH (n=2270)** | **PH (n=541)** |
| --- | --- | --- | --- |
| **Female gender** | 1876 (66.7%) | 1619 (71.3%) | 257 (47.5%) |
| **Median age (years)** | 48.0 [46.0-65.0] | 46.0 [36.0-56.0] | 57.0 [46.0-65.0] |
| **Ethnicity**  Black  White  Asian  Other  Mixed  Unknown | 163 (5.8%)  1246 (44.3%)  207 (7.4%)  150 (5.3%)  78 (2.8%)  912 (32.4%) | 97 (4.3%)  1031 (45.4%)  150 (6.6%)  114 (5.0%)  64 (2.8%)  760 (33.5%) | 66 (12.2%)  215 (39.7%)  57 (10.5%)  36 (6.7%)  14 (2.6%)  152 (28.1%) |
| **COVID-19 vaccination status**  Fully  Fully pre-COVID-19 infection | 1917 (68.2%)  318 (11.3%) | 1546 (68.1%)  277 (12.2%) | 371 (68.6%)  41 (7.6%) |
| **Employment status**  Employed & able to work  Employed & unable to work  Not employed  Retired  Unknown | 1505 (53.5%)  815 (29.0%)  203 (7.2%)  175 (6.2%)  113 (4.0%) | 1311 (57.8%)  640 (28.2%)  151 (6.67%)  98 (4.3%)  70 (3.1%) | 194 (35.9%)  175 (32.4%)  52 (9.6%)  77 (14.2%)  43 (8.0%) |
| **Disease characteristics at first assessment**  Recovered (>75% health)  FAS score  Number of symptoms (median [IQR])  EQ-5D score (median [IQR])  Percentage of best health, visual analogue scale (%)  Days since symptom onset (median [IQR]) | 366 (13.0%)  33 [22-38]  5 [1-7]  45 [25-66]  47 [29-70]  294 [91-393] | 244 (10.8%)  33.0 [26-39]  5.0 [3-8]  45 [30-60]  45 [30-60]  316 [177-533] | 122 (22.6%)  30.0 [22-38]  3.0 [1-7]  48.0 [25-66]  50.0 [29-70]  156.0 [91.0-393] |
| **Variant triggering infection**  Wild-type  Alpha  Delta  Omicron | 989 (35.2%)  967 (34.4%)  389 (13.8%)  466 (16.6%) | 864 (38.1%)  694 (30.6%)  272 (12.0%)  440 (19.4%) | 125 (23.1%)  273 (50.5%)  117 (21.6%)  26 (4.8%) |
| **Symptoms**  Fatigue  Breathlessness  Brain fog  Disturbed sleep  Chest Tightness  Arthralgia  Postural symptoms  Chest Pain  Focal weakness  Cough  Vertigo  Skin rash  No symptoms | 2136 (76.0%)  1738 (61.8%)  1439 (51.2%)  1144 (40.7%)  959 (34.1%)  958 (34.1%)  942 (33.5%)  799 (28.4%)  755 (26.9%)  662 (23.6%)  308 (11.0%)  276 (9.8%)  163 (5.8%) | 1809 (79.7%)  1414 (62.3%)  1258 (55.4%)  973 (42.9%)  826 (36.4%)  798 (35.2%)  825 (36.3%)  662 (29.2%)  639 (28.2%)  524 (23.1%)  268 (11.8%)  239 (10.5%)  85 (3.7%) | 327 (60.4%)  325 (59.9%)  181 (33.5%)  171 (31.6%)  133 (24.6%)  160 (29.6%)  117 (21.6%)  137 (25.3%)  116 (21.4%)  138 (25.5%)  40 (7.4%)  37 (6.8%)  78 (14.4%) |
| **Comorbidities**  Asthma  Hypertension  Diabetes mellitus, type 2  Migraine  Depression  Hyperthyroidism  Gastro-oesophageal reflux disease  Hypercholesterolaemia  Anxiety  Irritable bowel syndrome  Fibromyalgia  Chronic fatigue syndrome  Obesity  Polycystic ovarian syndrome  Osteoarthritis  Interstitial lung disease  Chronic obstructive pulmonary disease  Ischaemic heart disease  Iron deficiency anaemia  Diabetes mellitus, type 1 | 452 (16.1%)  347 (12.3%)  166 (5.9%)  234 (8.3%)  215 (7.7%)  168 (6.0%)  132 (4.7%)  129 (4.6%)  113 (4.0%)  103 (3.7%)  101 (3.6%)  76 (2.7%)  63 (2.2%)  62 (2.2%)  58 (2.1%)  55 (2.0%)  50 (1. 8%)  43 (1.5%)  37 (1.3%)  14 (0.5%) | 369 (16.3%)  198 (8.7%)  70 (3.1%)  212 (9.3%)  177 (7.8%)  132 (5.8%)  101 (4.5%)  79 (3.5%)  104 (4.6%)  94 (4.1%)  89 (3.9%)  72 (3.2%)  29 (1.3%)  53 (2.3%)  35 (1.5%)  13 (0.6%)  29 (1.28%)  22 (0.97%)  25 (1.1%)  11 (0.48%) | 83 (15.3%)  149 (27.5%)  96 (17.7%)  22 (4.1%)  38 (7.0%)  36 (6.7%)  31 (5.7%)  50 (9.2%)  9 (1.7%)  9 (1.7%)  12 (2.2%)  4 (0.7%)  34 (6.3%)  9 (1.7%)  23 (4.3%)  42 (7.8%)  21 (3.9%)  21 (3.9%)  12 (2.2%)  3 (0.6%) |

Legend: NH- non-hospitalised; PH- post-hospitalised

**Table S2.** **Baseline characteristics of Long COVID registry cohort at first Long COVID service assessment.**

|  | **Overall (N=4578)** | **Hospitalised (n=1213)** | **Non-hospitalised (n=3365)** |
| --- | --- | --- | --- |
| Median age, years [IQR] | 48.0 [37.5-59.0] | 58.0 [47.0-68.0] | 45.0 [35.0-55.0] |
| **Gender**  Male  Female  Unknown | 1751 (38.3%)  2825 (61.7%)  2 (0.0%) | 676 (55.73%)  537 (44.27%)  0 (0%) | 1075 (32.0%)  2288 (68.0%)  2 (0.1%) |
| **Age group**  18-24 years  25-34 years  35-44 years  45-54 years  55-64 years  65-74 years  75-84 years  85+ years | 146 (3.2%)  724 (15.8%)  997 (21.8%)  1074 (23.5%)  938 (20.5%)  433 (9.5%)  210 (4.6%)  56 (1.2%) | 16 (1.3%)  80 (6.6%)  153 (12.6%)  249 (20.5%)  309 (25.5%)  232 (19.1%)  133 (11.0%)  41 (3.4%) | 130 (3.9%)  644 (19.1%)  844 (25.1%)  825 (24.5%)  629 (18.7%)  201 (6.0%)  77 (2.3%)  15 (0. 5%) |
| **Ethnicity**  Black  White  Asian  Other  Mixed  Unknown | 496 (10.8%)  2955 (64.6%)  527 (11.5%)  390 (8.5%)  178 (3.9%)  32 (0.7%) | 213 (52.4%)  636 (14.2%)  173 (17.6%)  133 (4.0%)  49 (11.0%)  9 (0.7%) | 283 (68.9%)  2319 (10.5%)  354 (8.4%)  257 (3.8%)  129 (7.6%)  23 (0.7%) |
| **Index of Multiple Deprivation (IMD) quintile**  1  2  3  4  5  Unknown | 908 (19.8%)  1546 (33.8%)  1013 (22.1%)  688 (15.0%)  409 (8.9%)  14 (0.3%) | 345 (28.4%)  431 (35.5%)  235 (19.4%)  120 (9.9%)  79 (6.5%)  3 (0.3%) | 563 (16.7%)  1115 (33.1%)  778 (23.1%)  568 (16.9%)  330 (9.8%)  11 (0.3%) |
| **Full vaccination pre-COVID infection?**  Yes  No | 1323 (28.9%)  3255 (71.1%) | 133 (11.0%)  1080 (89.0%) | 1190 (35.4%)  2175 (64.6%) |
| **Long COVID service**  Median attendances, n [IQR]  Median duration of follow-up, days [IQR]*  Median time from 1 Jan 2020 to first assessment, years [IQR] | 1 [1-2]  0 [0-293]  1.8 [1.1-2.8] | 1 [1-2]  0 [0-147]  1.3 [1.1-2.1] | 1 [1-3]  0 [0-330]  2.0 [1.2-2.9] |

* between first and last attendance.

**Table S3: Follow-up and functional recovery in individuals with Long COVID with Patient-Reported Outcomes (PROMs) recorded at first and last assessment*.**

|  | **Non-Hospitalised** | **Hospitalised** | **Overall** | ***p*** |
| --- | --- | --- | --- | --- |
| Number (%) with ≥ 1 follow-up PROM | 1877 (71.2%) | 559 (58.7%) | 2436 (67.9%) | <0.001 |
| Number (%) who recovered over entire assessment period (followed-up individuals) | 338 (18.0%) | 117 (20.9%) | 455 (18.7%) | <0.001 |
| Median [IQR] time from symptom onset to functional recovery (days) (followed-up individuals) | 496 days [314-741] | 273 days [138-568] | 449 days [260-699] | <0.001 |
| Median [IQR] number of follow-up assessments to achieve functional recovery  (followed-up individuals) | 3 [2.0-4.0] | 2 [2.0-3.0] | 3 [2.0-4.0] | 0.01 |

*Functional recovery is defined as subjective attainment of 75% of best health. *P*-value (comparing hospitalised and non-hospitalised individuals) is by Mann-Whitney U test for continuous variables, and by chi-squared test for categorical variables.

**Table S4. Association between symptoms and risk of recovery in individuals with Long COVID (*n*=2811)*.**

|  | Hazard ratio (HR) | Standard. Error | 95% lower confidence interval (LCI) | 95% upper confidence interval (UCI) | *Z* | *p* |
| --- | --- | --- | --- | --- | --- | --- |
| Vaccination pre-COVID | 2.93 | 0.19 | 2.00 | 4.28 | 5.55 | <0.005 |
| Vertigo | 1.92 | 0.16 | 1.40 | 2.64 | 4.06 | <0.005 |
| Skin rash | 1.59 | 0.16 | 1.17 | 2.15 | 2.97 | <0.005 |
| Vaccination post-COVID | 1.34 | 0.12 | 1.05 | 1.71 | 2.33 | 0.02 |
| Male gender | 1.17 | 0.11 | 0.94 | 1.46 | 1.42 | 0.16 |
| Chest Pain | 1.12 | 0.14 | 0.86 | 1.47 | 0.85 | 0.40 |
| Chest Tightness | 1.09 | 0.13 | 0.84 | 1.41 | 0.62 | 0.54 |
| Cough | 1.03 | 0.13 | 0.80 | 1.33 | 0.24 | 0.81 |
| Brain fog | 0.85 | 0.13 | 0.66 | 1.10 | -1.23 | 0.22 |
| Disturbed sleep | 0.80 | 0.13 | 0.63 | 1.03 | -1.71 | 0.09 |
| Myalgia | 0.59 | 0.13 | 0.45 | 0.76 | -3.97 | <0.005 |
| Postural symptoms | 0.46 | 0.16 | 0.34 | 0.62 | -5.01 | <0.005 |
| Total FAS score | 0.37 | 0.06 | 0.33 | 0.42 | -15.32 | <0.005 |

*Cox proportional hazards model was stratified by hospitalisation status, breathlessness (symptom) and age group (see *Methods*).

**Table S5**. Factors associated with change in healthcare utilisation cost between Pre-assessment and Long COVID service periods in individuals with long COVID.

| **Covariates** | **Change in IP cost** | | **Change in ED cost** | | **Change in OP cost** | |
| --- | --- | --- | --- | --- | --- | --- |
|  | **Estimate (95% CI)** | **p value** | **Estimate (95% CI)** | **p value** | **Estimate (95% CI)** | **p value** |
| Age (years) | -1 (-17- 16) | 0.94 | 3 (1-4) | <0.001 | 2 (-4-8) | 0.47 |
| Gender |  |  |  |  |  |  |
| Male  Female | 0  254 (-249, 756) | 0.32 | 0 |  | 0 |  |
|  |  |  | 2 (-39-43) | 0.92 | 324 (141-507) | <0.001 |
| IMD quintile |  |  |  |  |  |  |
| 1  2  3  4  5 | 0  886 (206-1565)  494 (-253-1241)  871 (39-1703)  1065 (85-2045) | 0.01  0.20  0.04  0.03 | 0  7 (-48-63)  2 (-58-63)  -38 (-106-30)  -56 (-136-24) | 0.80  0.94  0.27  0.17 | 0  40 (-207- 288)  16 (-258-290)  85 (-224-395)  137 (-224-497) | 0.75  0.91  0.59  0.46 |
| Ethnicity |  |  |  |  |  |  |
| White  Asian or Asian British  Black or Black British  Mixed  Other ethnic groups | 0  252 (-517-1020)  -217 (-1017- 583)  706 (-551, 1962)  -321 (-1196-554) | 0.52  0.60  0.27  0.47 | 0  45 (-18-107)  -49 (-114-17)  20 (-83-122)  -26 (-97-46) | 0.16  0.14  0.71  0.48 | 0  71 (-210, 351)  -366 (-655- -77)  -427 (-889-35)  -57 (-373- 259) | 0.62  0.01  0.07  0.72 |
| Duration of follow up (days) | -0.54 (-1.38-0.30) | 0.21 | 0 (0-0.1) | 0.66 | 0 (0-1) | 0.03 |
| Time between long COVID onset and attendance at Long COVID service (weeks) | 4 (-3- 10) | 0.26 | 0 (-1-1) | 0.97 | 6 (3-9) | <0.001 |
| Time between start of pandemic and attendance at Long COVID service (years) | 209 (-124-542) | 0.22 | 117 (90-145) | <0.001 | 130 (-13-272) | 0.08 |
| Full COVID-19 vaccination pre-COVID-19 infection | 127 (-797-543) | 0.71 | -22 (-77-32) | 0.43 | 100 (-172- 371) | 0.47 |

Change in healthcare utilisation cost is calculated per-person cost per year in in-service period - per-person cost per year in pre-assessment period (more positive = smaller decrease from pre-assessment to in-service). Estimates and 95% CI are from separate multiple linear regression for IP, ED and OP cost change.

**Figure S1: Long COVID service structure at University College London Hospitals NHS Trust.**


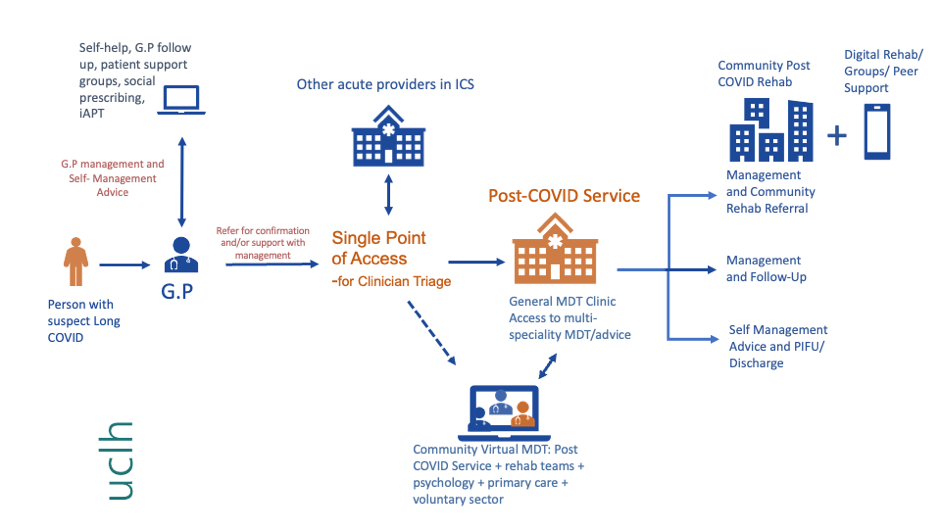


**Figure S2:** **COVID-19 variant and time to first presentation in individuals with Long COVID (*n*=2811).**


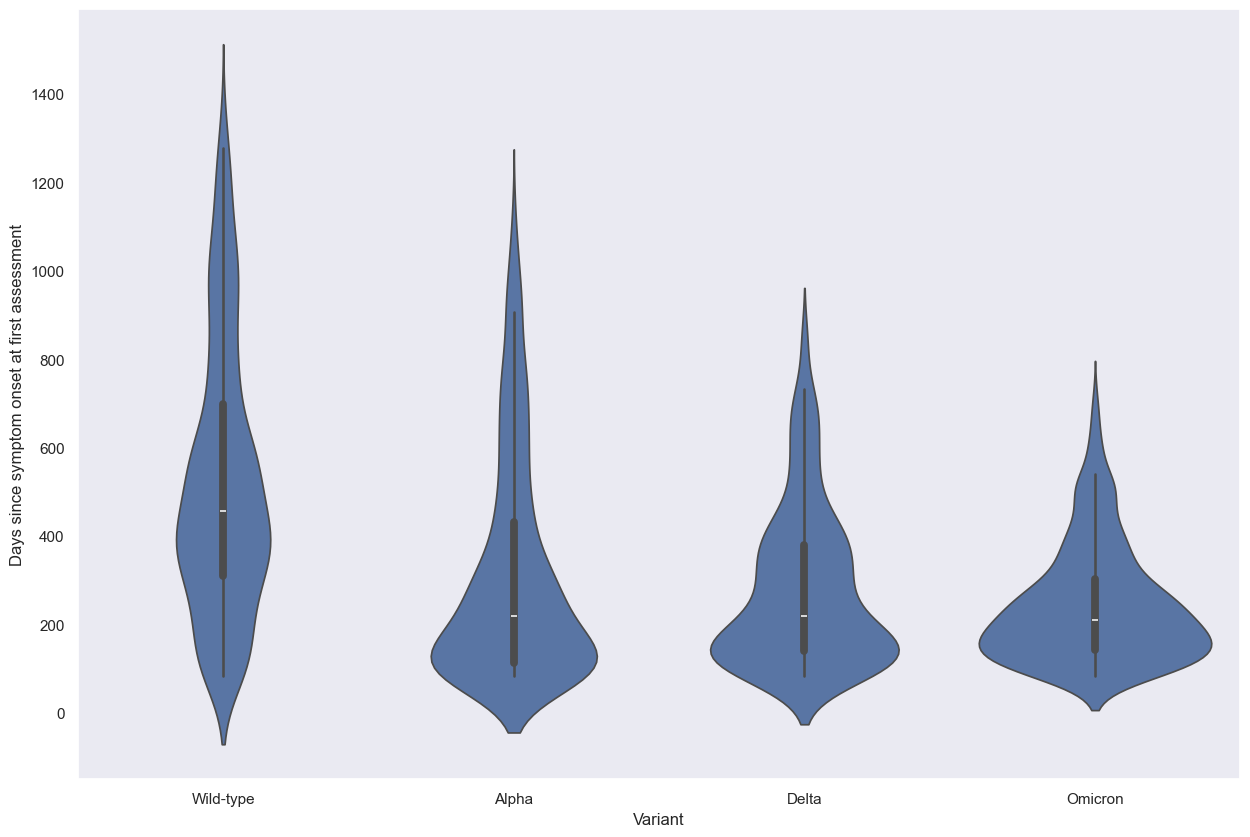


### **Figure S3:** **Proportion of individuals with Long COVID with symptoms at first presentation by triggering variant.**


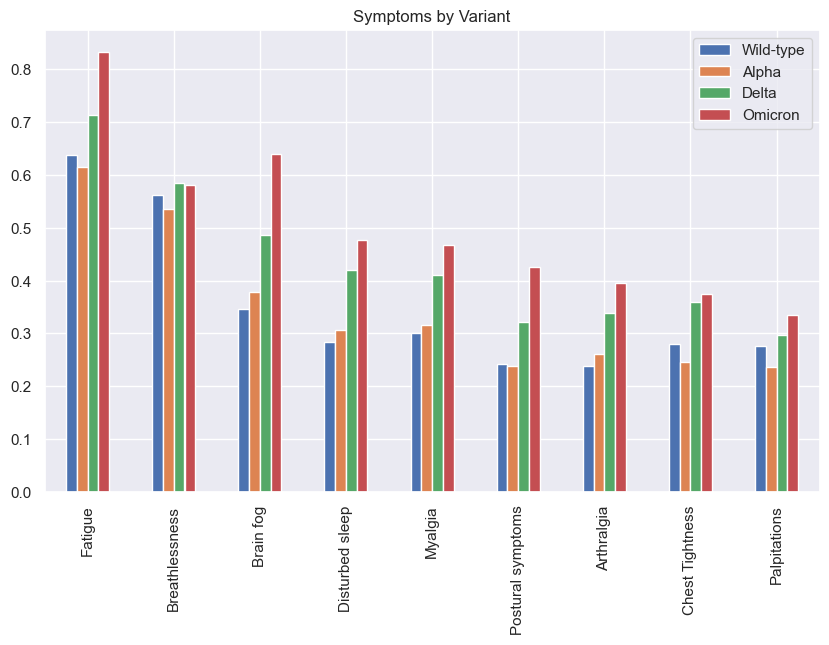


### Supplementary Figure 5: Cox proportional hazards regression in non-hospitalised individuals (*n*=2270).

### **Figure S4:** **Factors associated with recovery in non-hospitalised individuals with Long COVID in Cox proportional hazards regression (*n*=2270).**


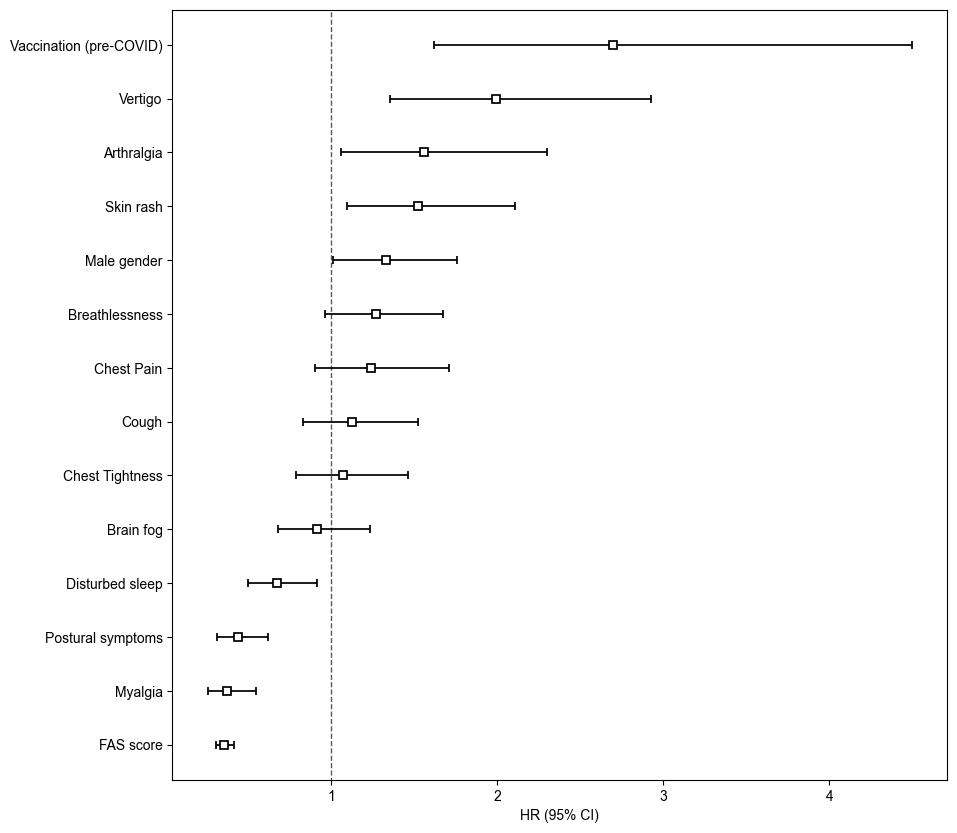


### **Figure S5: Factors associated with recovery in hospitalised individuals with Long COVID in Cox proportional hazards regression (n=541)*.**


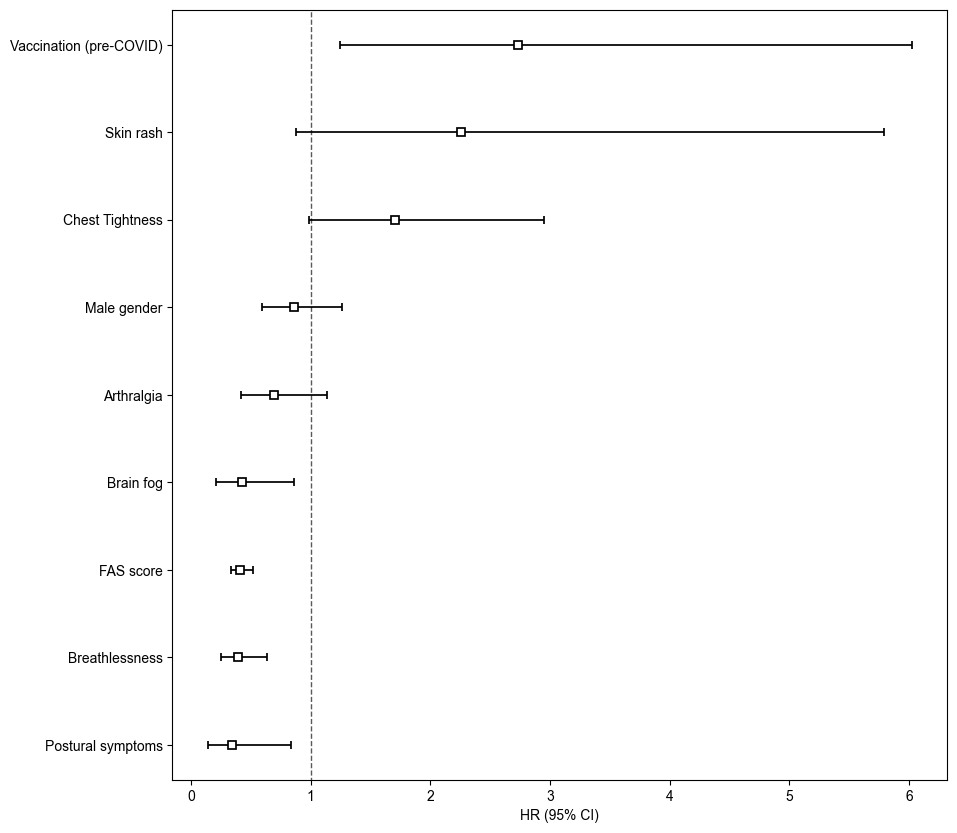

Supplement: online supplemental file 1 [file bmjopen-16-1-s001.docx]
