# Supplementary material for: Trajectory, healthcare utilisation and recovery in 3590 individuals with long covid: a 4-year prospective cohort analysis
Source: BMJ Open. 2026 Jan 14;16(1):e103884. doi: 10.1136/bmjopen-2025-103884 (PMC12815175; doi:10.1136/bmjopen-2025-103884)
Supplement: Supplementary file 2 [file bmjopen-2025-103884.reviewer_comments.pdf]

PEER REVIEW HISTORY

BMJ Open publishes all reviews undertaken for accepted manuscripts. Reviewers are asked to complete a checklist review form and are provided with free text boxes to elaborate on their assessment. These free text comments are reproduced below.

This paper was submitted to another journal from BMJ but declined for publication following peer review. The authors addressed the reviewers' comments and submitted the revised paper to BMJ Open. The paper was subsequently accepted for publication at BMJ Open.

ARTICLE DETAILS

Title (Provisional)

Trajectory, healthcare utilisation and recovery in 3590 individuals with Long COVID: a 4-year prospective cohort analysis.

Authors

Prashar, Jai; Hillman, Toby; Wall, Emma C; Sarna, Amanpreet; Mi, Emma; Bell, Robert; Sahota, Jagdeep; Zandi, Michael; McNamara, Patricia; Livingston, Rebecca; Gore, Rebecca; Lunken, Catherine; Bax, Elena; Nyam, Rachel; Rafie manzelat, Amir masood; Hishmeh, Lyth; Attree, Emily; Cone, Stephen; Banerjee, Amitava; Heightman, Melissa

---

VERSION 1 - REVIEW

**Reviewer**	**1**
**Name**	**Barker-Davies, Robert**
**Affiliation**	**DMRC Stanford Hall, Academic Department of Military Rehabilitation**
**Date**	**17-Apr-2025**
**COI**	**None**

Many thanks for the opportunity to review this paper from a well-respected group led by authors who have published widely on this topic. Their papers have included both qualitative and quantitative cohort data from long-COVID (LC) patients published in high impact factor journals and carry important messages for UK healthcare delivery. They have previously highlighted the need for integrated care allowing for a holistic approach and a focus on vocational rehabilitation. The findings of the current study are suitably novel and support the need for this approach in long term conditions and future pandemic preparedness. In particular, this large data set highlights the need for care of those that were not hospitalised with the acute illness. The main conclusions are perhaps a little open ended

and I believe could be refined in a way that continues to support the clear strategic messaging. I have detailed how I see these below and I hope the authors find the suggestions helpful. I would consider the changes minor and would be happy to look at a revised manuscript if the editors feel appropriate.

General points

The article is well written and mostly easily to follow except for a few specific areas detailed below.

The article will be of interest to healthcare professionals across disciplines, patients and policymakers including employers given the median age of 50. Given the size of the sample and well-presented demographic data the pragmatic study design is reflective of the UK population. As noted by the authors the lack of a matched control group is a limitation particularly when using the EQ5-D VAS to define recovery status as this is a very open question for which a score <75 could represent morbidity from unrelated conditions.

Abstract

Is representative of the full-text.

There is no “what is known on this topic”/”what the study adds” box as per the BMJ author guidelines.

Introduction

The introduction does not end with a clear testable hypothesis which is fine given that post-COVID syndrome/LC is still a novel disease and this cohort dates back to the onset of the pandemic. However, given the multiple outcome measures and variability in follow up points the conclusion on p14 line 14 “with only one-third achieving functional recovery over 4 years in this cohort” is a little misleading. Explained further below.

Results

Four years is not a variable in the data from which the main conclusions have been drawn. The longevity of the service (4 years) is commendable, and the 4-year timeline is noteworthy due to the changes that have occurred during that time, COIVD variants, introduction of vaccination, change in public perception etc. The number of participants in the title carries weight and conveys that this is a substantial sample. However, the main conclusion seems to be based on n=2085 p.10 line 22 – 58% of the sample (table 2). It is likely that patients who chose not to attend follow-up did so because they did not require further care and score >75

on the EQ5-D VAS. Do the authors have any data on reasons given for not attending follow-up (STROBE checklist point 13.b)? I looked for the total number of patients at follow-up and % with EQ5d VAS> 75 as presented in table 1 for the baseline (n=804, 22.4%). This is represented graphically and in adjusted form in figure 3 but the data appears to be in supplemental table S3. The proportion in table S3 is 455 recovered to >75 (18.7% of n=2436). So it's unclear where n=1198 (33.4%) recovered to >75% during follow-up comes from. It's implied from the text this data is in table 3 (p.10 ln 34) but that along with the median onset to recovery figure (202 (94-468) days) I can't seem to find. I would rectify this by removing "4-years" from the title and add further clarity throughout the manuscript on the various follow up points as follows.

The nomenclature of the various follow-up points would benefit from further clarity eg P9. Ln 27 reads median follow-up was 136 days – does this mean the first follow-up point where n=3590 on figure 1? Perhaps standadise how the follow-up points are referred to in the text and align to figure 1 for clarity i.e. consistently refer to say "baseline" (where n=5253) rather than interchange with "first assessment". Then "First follow-up" where n=3590 and "final follow-up" where n=2085-2811 (depending on outcomes completed).

In another example the "final follow-up" on page 10 line 18 is referred to as "last recorded visit" and again line 27 "last assessment in followed-up individuals" and then line 30 – "during follow-up". I would standardise these so it's clear to the reader that they are the same timepoint. The 502 days median figure for the timing of the "final follow-up" quoted in the abstract results section is not in the main text of the article, though does appear at the bottom of table 1. Given the large range 251-825 days and the impact this may have on the main conclusions it may be useful to see a graphical representation of the spread of this data. Something similar to figure S2 which is time to "first presentation" but for "final follow-up" and included in the main document. This would be informative because previous large cohort post-COVID studies have found no/little improvement between 6-12 months (Evans 2022, Lancet respiratory medicine)(Huang 2021, Lancet) but subsequent significant improvement at two years (Huang 2022, Lancet Respiratory Medicine). This is not to suggest that the main conclusions of this paper are not correct in calling for ongoing service provision but the proportion of patients appearing to have enduring problems may appear overrepresented in the way the first sentence of the abstract conclusion section (p4 line 52) is not time bound (eg at a median follow-up of either 16months (502days (p4, line 35) or 7 months (202 days, p4 line 41)). In combination with the title containing "4-years' at first glance it could be taken only 1/3 of patients have recovered at 4 years follow-up. In the abstract conclusion I would add a time frame on recovery eg "at x months follow-up". The way this is currently stated implies more permanence of the condition.

Including the median timepoint of "baseline" and "first follow-up" and "final follow-up" on figure 1 would also add clarity to the above.

With regards to the FAS results could the change in FAS from 33 to 31 p.10 line 18 be presented alongside a Minimally Clinically Important Difference statistic from the literature or an effect size be calculated? I think this would help make the distinction between a statistically significant decrease (largely due to the size of the sample, table 2) but actually quite a small overall effect. This would support the main findings of the need for ongoing care. Also, as fatigue is the commonest symptom in LC it would deserve the emphasis. From the literature and the current study the main issue requiring ongoing care is managing fatigue.

Discussion

P.11 line 38 “short and long-term functional recovery are very low” need qualifying as above eg “to a median of x months follow-up”.

P.12 Line 48 “further studies with longer follow-up are required”. As mentioned above further comparison to longer follow-up studies, the potential for recovery and when that is likely to occur is worth discussion at this point. Why is “early care effective”? It is worth exploring the mechanisms (see below).

P.13 Line 20 “Can reduce morbidity” some exploration of the underlying mechanisms addressing the main symptoms and findings of the paper which is fatigue would enlighten the reader further here eg Parker’s paper (M Sivan’s group) demonstrating the positive effect of pacing on post exertional malaise. Parker M, Sawant HB, Flannery T, Tarrant R, Shardha J, Bannister R, Ross D, Halpin S, Greenwood DC, Sivan M. Effect of using a structured pacing protocol on post-exertional symptom exacerbation and health status in a longitudinal cohort with the post-COVID-19 syndrome. J Med Virol. 2023 Jan;95(1):e28373. doi: 10.1002/jmv.28373. PMID: 36461167; PMCID: PMC9878088. I think this would give a helpful, positive message that these patients can experience sustainable functional improvements when well-managed. It would also support the statement on p12 line 48 “Early intervention may be associated with better outcomes”.

P.13 Line 50 Limitations: Given the lack of control group it may be worth noting that questionnaire data of this type can overestimate prevalence particularly when the questionnaire is completed within a specialist service rather than a general population which may result in a contextual/framing effect. The Scott 2023 paper below discusses the concept in relation to healthcare workers and mental health screening during the COVID pandemic. Although the main outcome measures differ with the Scott paper’s focus on mental health

there is considerable overlap among questions of this type. For example, within the FAS (I have problems starting things) compared with GHQ-12 questions (eg Couldn't overcome difficulties).

Prevalence of post-traumatic stress disorder and common mental disorders in health-care workers in England during the COVID-19 pandemic: a two-phase cross-sectional study Scott, Hannah R et al. The Lancet Psychiatry, Volume 10, Issue 1, 40 - 49

**Reviewer**	**2**
**Name**	**Reviewer 2**
**Affiliation**	
**Date**	**17-Apr-2025**
**COI**	

Comments not published pending reviewer's consent.

**Reviewer**	**3**
**Name**	**Reviewer 3**
**Affiliation**	
**Date**	**17-Apr-2025**
**COI**	

Comments not published pending reviewer's consent.

**Reviewer**	**4**
**Name**	**Reviewer 4**
**Affiliation**	
**Date**	**17-Apr-2025**
**COI**	

Comments not published pending reviewer's consent.

VERSION 1 - AUTHOR RESPONSE

Reviewer: 1

Please confirm that you understand and consent to the above terms and conditions.: I consent to the publication of this review

Please enter your name: Robert M Barker-Davies

Job Title: Senior Lecturer in Sport, Exercise and Rehabilitation Medicine

Institution: Academic Department of Military Rehabilitation, Defence Medical Services

Comments:
Summary

Many thanks for the opportunity to review this paper from a well-respected group led by authors who have published widely on this topic. Their papers have included both qualitative and quantitative cohort data from long-COVID (LC) patients published in high impact factor journals and carry important messages for UK healthcare delivery. They have previously highlighted the need for integrated care allowing for a holistic approach and a focus on vocational rehabilitation. The findings of the current study are suitably novel and support the need for this approach in long term conditions and future pandemic preparedness. In particular, this large data set highlights the need for care of those that were not hospitalised with the acute illness. The main conclusions are perhaps a little open ended and I believe could be refined in a way that continues to support the clear strategic messaging. I have detailed how I see these below and I hope the authors find the suggestions helpful. I would consider the changes minor and would be happy to look at a revised manuscript if the editors feel appropriate.

We thank the reviewer for their kind comments on our work, particularly the novel nature of the data. We address their comments and suggestions below.

General points

The article is well written and mostly easily to follow except for a few specific areas detailed below.

The article will be of interest to healthcare professionals across disciplines, patients and policymakers including employers given the median age of 50. Given the size of the sample and well-presented demographic data the pragmatic study design is reflective of the UK population. As noted by the authors the lack of a matched control group is a limitation particularly when using the EQ5-D VAS to define recovery status as

this is a very open question for which a score <75 could represent morbidity from unrelated conditions.

We agree that this is a key limitation of our data. While an EQ-5-D VAS of <75 could represent morbidity extraneous to long-COVID related illness, this is a widely noted challenge of using visual analogue symptom scales. We make the assumption that such effects would be evenly distributed within groups at baseline and further examine change in EQ-5-D VAS and FAS scores in order to assess improvement independent of baseline functional status.

Abstract

Is representative of the full-text.

There is no "what is known on this topic"/"what the study adds" box as per the BMJ author guidelines.

Thank you for highlighting this. This has now been included.

Introduction

The introduction does not end with a clear testable hypothesis which is fine given that post-COVID syndrome/LC is still a novel disease and this cohort dates back to the onset of the pandemic. However, given the multiple outcome measures and variability in follow up points the conclusion on p14 line 14 "with only one-third achieving functional recovery over 4 years in this cohort" is a little misleading. Explained further below.

We agree that this description could be improved to better represent the structure of the data. This has been changed to 'evidence of significant and prolonged functional impairment in this cohort'.

Results

Four years is not a variable in the data from which the main conclusions have been drawn. The longevity of the service (4 years) is commendable, and the 4-year timeline is noteworthy due to the changes that have occurred during that time, COIVD variants,

introduction of vaccination, change in public perception etc. The number of participants in the title carries weight and conveys that this is a substantial sample.
However, the main conclusion seems to be based on n=2085 p.10 line 22 – 58% of the sample (table 2). It is likely that patients who chose not to attend follow-up did so because they did not require further care and score >75 on the EQ5-D VAS.

Thank you for highlighting this. The *n*=2085 refers to the cohort of individuals for whom at least one follow-up overall EQ-5-D score was available, rather than the cohort for whom the regression analysis is based (those for whom at least one E-Q-5D VAS was available, as well as FAS and symptom data, *n*=2811). The latter includes those with one assessment, given that *t*=0 in our survival analysis is symptom onset, and patients often recovered prior to first assessment.

Do the authors have any data on reasons given for not attending follow-up (STROBE checklist point 13.b)? I looked for the total number of patients at follow-up and % with EQ5d VAS> 75 as presented in table 1 for the baseline (n=804, 22.4%). This is represented graphically and in adjusted form in figure 3 but the data appears to be in supplemental table S3. The proportion in table S3 is 455 recovered to >75 (18.7% of n=2436). So it's unclear where n=1198 (33.4%) recovered to >75% during follow-up comes from. It's implied from the text this data is in table 3 (p.10 ln 34) but that along with the median onset to recovery figure (202 (94-468) days) I can't seem to find. I would rectify this by removing "4-years" from the title and add further clarity throughout the manuscript on the various follow up points as follows.

Follow-up in this study was based on clinical need, as determined by the clinician seeing the patient. Unfortunately, we do not have granular data on the specific justification for this in every case as these are routine clinical data. The number in Table S3 refers to followed-up individuals only, whereas *n*=1198 refers to all individuals; the discrepancy is again explained by the fact that patients were followed up based on clinical need rather than to a protocol. The figures to which the reviewer refers are found in Table 1 (bottom panel).

The nomenclature of the various follow-up points would benefit from further clarity eg P9. Ln 27 reads median follow-up was 136 days – does this mean the first follow-up point where n=3590 on figure 1? Perhaps standardise how the follow-up points are referred to in the text and align to figure 1 for clarity i.e. consistently refer to say "baseline" (where n=5253) rather than interchange with "first assessment". Then "First

follow-up" where n=3590 and "final follow-up" where n=2085-2811 (depending on outcomes completed).

The median follow-up of 136 days described includes all individuals, including those discharged at first assessment. While *n*=5253 were seen in the service over the entire period, the minimum number with 'complete' data (see Figure 1) at first assessment, and included in this study, was 3590. We use 'baseline' and 'first assessment' interchangeably; however, we agree that this may create confusion and have replaced all instances of 'baseline characteristics' with 'characteristics at first assessment' to avoid this.

In another example the "final follow-up" on page 10 line 18 is referred to as "last recorded visit" and again line 27 "last assessment in followed-up individuals" and then line 30 – "during follow-up". I would standardise these so it's clear to the reader that they are the same timepoint.

We agree; the text now refers only to 'last assessment'.

The 502 days median figure for the timing of the "final follow-up" quoted in the abstract results section is not in the main text of the article, though does appear at the bottom of table 1.

This has now been added to the *Results*.

Given the large range 251-825 days and the impact this may have on the main conclusions it may be useful to see a graphical representation of the spread of this data. Something similar to figure S2 which is time to "first presentation" but for "final follow-up" and included in the main document. This would be informative because previous large cohort post-COVID studies have found no/little improvement between 6-12 months (Evans 2022, Lancet respiratory medicine)(Huang 2021, Lancet) but subsequent significant improvement at two years (Huang 2022, Lancet Respiratory Medicine). This is not to suggest that the main conclusions of this paper are not correct in calling for ongoing service provision but the proportion of patients appearing to have enduring problems may appear overrepresented in the way the first sentence of the abstract conclusion section (p4 line 52) is not time bound (eg at a median follow-up of either

16months (502days (p4, line 35) or 7 months (202 days, p4 line 41)). In combination with the title containing "4-years' at first glance it could be taken only 1/3 of patients have recovered at 4 years follow-up. In the abstract conclusion I would add a time frame on recovery eg "at x months follow-up". The way this is currently stated implies more permanence of the condition.

We agree that it is important to clarify the length of follow-up and adjust our conclusions accordingly, particularly in view of the literature you have cited. We have removed the '4 year' timeframe from the title. Adding a specific timeframe to the conclusion may be misleading in that follow-up was bound to clinical need in this real-world study. As suggested, we have produced a figure to graphically represent the spread of time from initial assessment to final follow-up; this can be found in Figure S6.

Including the median timepoint of "baseline" and "first follow-up" and "final follow-up" on figure 1 would also add clarity to the above.

'Baseline' and 'first assessment' are equivalent, with the latter now being used in the text for clarity as per your suggestion above. Median time from symptom onset to 'final follow-up' is represented in Table 1 ('initial assessment to discharge') and graphically in Figure S6 as above.

With regards to the FAS results could the change in FAS from 33 to 31 p.10 line 18 be presented alongside a Minimally Clinically Important Difference statistic from the literature or an effect size be calculated? I think this would help make the distinction between a statistically significant decrease (largely due to the size of the sample, table 2) but actually quite a small overall effect. This would support the main findings of the need for ongoing care. Also, as fatigue is the commonest symptom in LC it would deserve the emphasis. From the literature and the current study the main issue requiring ongoing care is managing fatigue.

We agree with the reviewer. Given the paucity of studies in this area, we were not able to identify a Minimally Clinically Important Difference for FAS in LC in the literature (although some studies exist in other conditions). We have calculated Cohen's *d* which, as expected, indicates a small effect size for FAS and EQ-5-D VAS between first and last assessment – supporting the persistence of fatigue in association with overall morbidity. This can be found in Table 2 and the main text.

Discussion

P.11 line 38 "short and long-term functional recovery are very low" need qualifying as above eg "to a median of x months follow-up".

Added ***'very low over the median follow-up period of 502 days'.***

P.12 Line 48 "further studies with longer follow-up are required". As mentioned above further comparison to longer follow-up studies, the potential for recovery and when that is likely to occur is worth discussion at this point. Why is "early care effective"? It is worth exploring the mechanisms (see below).

Thanks for this comment. We specifically say that "Early intervention may be associated with better outcomes" because trial evidence is lacking but there are observational data which seem to support early intervention, e.g.

More successful "return to work":

Nagra G, Ezeugwu VE, Bostick GP, Branton E, Dennett L, Drake K, Durand-Moreau Q, Guptill C, Hall M, Ho C, Hung P, Khan A, Lam GY, Nowrouzi-Kia B, Gross D. Return-to-work for People Living with Long COVID: A Scoping Review of Interventions and Recommendations https://www.medrxiv.org/content/10.1101/2024.12.10.24318765v1.full

Therefore, any discussion of mechanism is speculative at best. However, we have added to the sentence to reflect potential mechanism.

P.13 Line 20 "Can reduce morbidity" some exploration of the underlying mechanisms addressing the main symptoms and findings of the paper which is fatigue would enlighten the reader further here eg Parker's paper (M Sivan's group) demonstrating the positive effect of pacing on post exertional malaise. Parker M, Sawant HB, Flannery T, Tarrant R, Shardha J, Bannister R, Ross D, Halpin S, Greenwood DC, Sivan M. Effect of using a structured pacing protocol on post-exertional symptom exacerbation and health status in a longitudinal cohort with the post-COVID-19 syndrome. J Med Virol. 2023 Jan;95(1):e28373. doi: 10.1002/jmv.28373. PMID: 36461167; PMCID: PMC9878088. I think this would give a helpful, positive message that these patients can experience sustainable functional improvements when well-managed. It would also

support the statement on p12 line 48 “Early intervention may be associated with better outcomes”.

We agree that addressing fatigue as a significant component of long COVID-associated morbidity may benefit from further comment. We have cited the suggested paper (a prospective pre-post study without a control group), but preface this with the fact that the evidence for rehabilitative or other interventions in managing long-COVID related fatigue remains scarce in the literature overall – reinforcing the call for further research.

P.13 Line 50 Limitations: Given the lack of control group it may be worth noting that questionnaire data of this type can overestimate prevalence particularly
when the questionnaire is completed within a specialist service rather than a general population which may result in a contextual/framing effect. The Scott 2023 paper below discusses the concept in relation to healthcare workers and mental health screening during the COVID pandemic. Although the main outcome measures differ with the Scott paper’s focus on mental health there is considerable overlap among questions of this type. For example, within the FAS (I have problems starting things) compared with GHQ-12 questions (eg Couldn’t overcome difficulties).

Prevalence of post-traumatic stress disorder and common mental disorders in health-care workers in England during the COVID-19 pandemic: a two-phase cross-sectional study
Scott, Hannah R et al. The Lancet Psychiatry, Volume 10, Issue 1, 40 - 49

We agree that the absence of a control group would almost certainly lead to a framing effect; as such, it is important that our findings are taken in the context of the specialist service in which they were derived. We cannot specifically infer the relative prevalence of symptoms in the general population from this work (as Scott *et al* refer to in their prevalence study) and have strengthened this point in the *Limitations* as a result.

VERSION 2 – REVIEW

**Reviewer**	**1**
**Name**	**Barker-Davies, Robert**

**Affiliation**	**DMRC Stanford Hall, Academic Department of Military Rehabilitation**
**Date**	**10-Oct-2025**
**COI**	

Many thanks to the authors for the point-by-point response to my review. I am satisfied that all points raised, which I considered minor, have been suitably addressed and reflected in the latest version of the manuscript.

**Reviewer**	**5**
**Name**	**Dack, Kyle**
**Affiliation**	**University of Bristol, MRC Integrative Epidemiology Unit**
**Date**	**02-Aug-2025**
**COI**	**None**

Statistical review summary:

The design and analysis of this study are generally strong, and results are presented and interpreted clearly. However, the methods section lacks clarity in key areas (study dates, followup time, variable selection) and would benefit from reorganizing into more distinct sections (STROBE may help this). One statistical concern I do have is that the study inclusion dates and follow-up dates seem to be the same, which means that late diagnosed individuals lack time to express the outcomes which could skew certain statistics, and it is unclear how this was handled (e.g. minimum followup time).

Abstract:

1. Generally clear and comprehensive, including all essential elements for abstract readers. However, the result reported on line 42 include the outcome “shorter time to recovery”. From the methods and results, this appears to be the HR of recovering to 75% of best health. The interpretation of it as reflecting shorter recovery time is not used in the rest of the paper, and I suggest rephrasing for consistency between abstract and main paper. As written, it could be misinterpreted as a direct comparison of recovery duration or speed.

Introduction:

2. Clear and meeting the STROBE guidelines – no suggestions.

Methods:

3. Study population inclusion dates: There are a few different dates which were used for including patients. The general inclusion dates appear to be if patients were diagnosed between 1st April 2020 to 31st March 2024. There is also registry-based cohort with dates of

11th May 2020 and 30th November 2023, and the HCRU analysis uses dates of 1st Jan 2018 to 28th February 2024. I could not follow if inclusion in the latter two cohorts is also based on diagnosis date? It may be clearer to readers if there was first a brief overview of the different cohorts in this study, followed by a summary of the study dates used for each (and what those study dates mean). E.g. An additional paragraph in “study design”.

4. Assuming the three study periods described above are all based on diagnosis date, was there a minimum follow-up time required for inclusion? If not, late-diagnosed patients could have insufficient observation time for outcomes to be expressed, such as an individual diagnosed in January 2024 being classified as not yet recovered, whereas an individual diagnosed earlier in the study has had more time for this outcome to occur. This would introduce differential follow-up across participants and may bias estimates of recovery time or rates. I could not find information about how this was handled, e.g. through censoring, time-to-event methods, or sensitivity analyses applying a minimum follow-up duration - and suggest this is clarified.

5. Outcomes: The STROBE guidelines recommend defining each outcome, in terms of what it represents, and how it was measured and coded. There are some details in the methods, and in the supplementary materials, but it is dispersed and not easy to find all information. Given that the outcomes are of central importance to understanding the study, I suggest these would be better suited in the main document, under a “data”, “variables” or other subheading.

6. Missing data: It is not clear from the description whether imputation of follow-up PROMs was applied only to individuals with partial missingness while still under follow-up, or also to those lost to follow-up entirely.

7. The methods of variable selection are currently in the supplementary methods, but this is a critical element of the paper and I suggest keeping it in the statistical analysis section. Additionally, transparency could be strengthened by stating the specific variables which were added via feature selection, and which were added due to clinical importance.

8. HCRU outcomes are currently defined under the “data analysis” subheading. I would suggest moving this to a separate subheading where outcomes are defined, so that the data analysis section is focused solely on statistical procedures.

9. HCRU models: the variable selection does not use the same methodology as the primary outcome. What was the rationale, and how were the variables selected? If it was evidence-based to minimise confounding, I recommend directly stating this to ensure readers understand that this was considered.

10. This is touched upon in previous comments but the follow-up dates are a little unclear – I may have missed it but I cannot find information about when the primary outcome was measured. It looks as if symptom burden was also measured during follow-up, but it is not clear when this was, and it impacts how well the results can be interpreted.

Results

11. “there were no significant differences compared with the cohort included in Cox regression models (n=2811)” suggest making it clearer what is being compared “no significant differences between those with baseline characteristics, and those with adequate follow-up to be included in the Cox’s regression cohort” (if I have interpreted these correctly).

12. The median reported FAS was 31 at the last assessment. It would be helpful to readers to understand how long this tended to be from the first assessment, e.g. the median number of days and/or assessments between the two.

13. Tables/figures: Clearly presented, and no concerns/suggestions other than those already mentioned in regards to the methods.

Discussion

14. Results are interpreted reasonably. There is a clear discussion of clinical implications and limitations. However, the impact of late-diagnosed patients should be discussed. I could not find aspects of the methodology which address these patients, and there is a risk that including those with a lack of adequate follow-up time could skew certain statistics. This could be explored through sensitivity analysis but with so much analysis, this may be challenging. The authors could add it as a limitation and highlight if any particular results could be vulnerable to this issue.

VERSION 2 - AUTHOR RESPONSE

Reviewer 1	
Many thanks to the authors for the point-by-point response to my review. I am satisfied that all points raised, which I considered minor, have been suitably addressed and reflected in the latest version of the manuscript.	Thank you for your response. We are pleased that we have suitably addressed the points made.
Reviewer 5	
Statistical review summary:<br>The design and analysis of this study are generally strong, and results are presented and interpreted clearly. However, the methods section lacks clarity in key areas (study dates, followup time, variable selection) and would benefit from reorganizing into more distinct sections (STROBE may help this). One statistical concern I do have is that the study inclusion dates	We thank the reviewer for this supportive comment.<br><br>In regard to the methods section, we have re-organised the Methods to better fit the STROBE guidelines with reference to variable selection and follow-up (under study design).<br><br>Regarding follow up timing in the manuscript: referral to the service was contingent on a disease duration of at least 6 weeks or more

and follow-up dates seem to be the same, which means that late diagnosed individuals lack time to express the outcomes which could skew certain statistics, and it is unclear how this was handled (e.g. minimum followup time).	(see ~~Methods~~). We report the time to first assessment was median 236 days; see Table 1). Based on our clinical experiences of patient trajectories in long COVID, it is clinically very unlikely that patient symptom characteristics will change after this time [Ref Heightman et al BMJ Open 2022).<br><br>We agree that clarity on this point is important, and have noted this as a limitation in the Discussion.
Abstract:<br>1. Generally clear and comprehensive, including all essential elements for abstract readers. However, the result reported on line 42 include the outcome "shorter time to recovery". From the methods and results, this appears to be the HR of recovering to 75% of best health. The interpretation of it as reflecting shorter recovery time is not used in the rest of the paper, and I suggest rephrasing for consistency between abstract and main paper. As written, it could be misinterpreted as a direct comparison of recovery duration or speed.<br><br>Introduction:<br>2. Clear and meeting the STROBE guidelines – no suggestions.	Thank you for your comments.<br><br>We agree that clarification that this does not refer to direct duration or speed of recovery would be helpful, and have adjusted the wording accordingly (Abstract > Results, page 4).
3. Study population inclusion dates: There are a few different dates which were used for including patients. The general inclusion dates appear to be if patients were diagnosed between 1st April 2020 to 31st March 2024. There is also registry-based cohort with dates of 11th May 2020 and 30th November 2023, and the HCRU analysis uses dates of 1st Jan 2018 to 28th February 2024. I could not follow if inclusion in the latter two cohorts is also based on diagnosis date? It may be clearer to readers if there was first a brief overview of the different cohorts in this study, followed by a summary of the	We thank the reviewer for highlighting this issue. This interpretation is largely correct; the registry-based cohort included patients presenting from April 2020, but we made use of healthcare utilisation data from Jan 2018 onwards to allow us to build the 'pre-COVID' healthcare utilisation period. Presenting date, rather than diagnosis date was used in the cohort selection logic to enable the analyses.<br><br>We have clarified this by moving a paragraph from 'data sources' to 'study population' and adding further detail.

study dates used for each (and what those study dates mean). E.g. An additional paragraph in "study design".	
4. Assuming the three study periods described above are all based on diagnosis date, was there a minimum follow-up time required for inclusion? If not, late-diagnosed patients could have insufficient observation time for outcomes to be expressed, such as an individual diagnosed in January 2024 being classified as not yet recovered, whereas an individual diagnosed earlier in the study has had more time for this outcome to occur. This would introduce differential follow-up across participants and may bias estimates of recovery time or rates. I could not find information about how this was handled, e.g. through censoring, time-to-event methods, or sensitivity analyses applying a minimum follow-up duration - and suggest this is clarified.	Please see our response above. Based on the design of the service, time to first assessment and our clinical judgement, the strong expectation is that all patients would have expressed these characteristics prior to first assessment (see Figure 2).<br><br>We have clarified this accordingly.
5. Outcomes: The STROBE guidelines recommend defining each outcome, in terms of what it represents, and how it was measured and coded. There are some details in the methods, and in the supplementary materials, but it is dispersed and not easy to find all information. Given that the outcomes are of central importance to understanding the study, I suggest these would be better suited in the main document, under a "data", "variables" or other subheading.	We thank the reviewer for this comment. We agree that this would be useful for clarity, and have concentrated this information in the main manuscript under a heading called 'variables' as suggested.
6. Missing data: It is not clear from the description whether imputation of follow-up PROMs was applied only to individuals with partial missingness while still under follow-up, or also to those lost to follow-up entirely.	The imputation procedure was applied to those with partial missingness.<br><br>Our data missingness was due to absence of recorded PROM dates (e.g. data recording/administrative errors rather than due to loss to follow-up).

	We have added a sentence to the text (Methods > Symptom burden, page 9 line number) to clarify.
7. The methods of variable selection are currently in the supplementary methods, but this is a critical element of the paper and I suggest keeping it in the statistical analysis section. Additionally, transparency could be strengthened by stating the specific variables which were added via feature selection, and which were added due to clinical importance.	We thank the reviewer for this important comment.<br>We agree that this is important information; while we feel that the technical detail is of less interest to the general medical reader, and for brevity, we kept an overall view of variable selection in the main methods and retained detailed methodology in the supplementary for a more specialist audience.<br><br>We have also indicated variables added due to clinical importance in the Supplementary Material.
8. HCRU outcomes are currently defined under the “data analysis” subheading. I would suggest moving this to a separate subheading where outcomes are defined, so that the data analysis section is focused solely on statistical procedures.	We thank the reviewer for this helpful suggestion. We have moved this description to the 'Variables’ subheading as suggested.
9. HCRU models: the variable selection does not use the same methodology as the primary outcome. What was the rationale, and how were the variables selected? If it was evidence-based to minimise confounding, I recommend directly stating this to ensure readers understand that this was considered.	The variable selection for the HCRU analysis was performed based on clinical judgement of the consultant physicians in the UCLH Long COVID clinic; the available variables on the national data analysis platform were largely limited to sociodemographic factors.<br><br>We have included this in the Methods.
10. This is touched upon in previous comments but the follow-up dates are a little unclear – I may have missed it but I cannot find information about when the primary outcome was measured. It looks as if symptom burden was also measured during follow-up, but it is not clear when this was, and it impacts how well the results can be interpreted.	Please see above. The primary outcome was measured at each assessment.<br><br>FAS, EQ5D and symptom burden were measured systematically through electronic health records by either the patient before the appointment or the clinician at each follow-up assessment with varying levels of data completeness (see Table 2). We have

	clarified this in the Methods, under the Variables subheading.
11. "there were no significant differences compared with the cohort included in Cox regression models (n=2811)" suggest making it clearer what is being compared "no significant differences between those with baseline characteristics, and those with adequate follow-up to be included in the Cox's regression cohort" (if I have interpreted these correctly).	Thank you for this comment; your interpretation is correct and we have adjusted the wording as suggested to improve clarity for the reader.
12. The median reported FAS was 31 at the last assessment. It would be helpful to readers to understand how long this tended to be from the first assessment, e.g. the median number of days and/or assessments between the two.	Thank you for your comment. This information is contained in Table 1: (median time from initial assessment to discharge in days (136 days [IQR 0-346 days])
13. Tables/figures: Clearly presented, and no concerns/suggestions other than those already mentioned in regards to the methods.	We thank the reviewer for the supportive comments on our tables and figures.
14. Results are interpreted reasonably. There is a clear discussion of clinical implications and limitations. However, the impact of late-diagnosed patients should be discussed. I could not find aspects of the methodology which address these patients, and there is a risk that including those with a lack of adequate follow-up time could skew certain statistics. This could be explored through sensitivity analysis but with so much analysis, this may be challenging. The authors could add it as a limitation and highlight if any particular results could be vulnerable to this issue.	Many thanks for highlighting this point. As discussed above, we made use of the considerable median time from symptom onset to first assessment (236 days) to mitigate against this issue. We do not anticipate that phenotypic characteristics were likely to emerge after this point.<br><br>We have added this point to the Discussion in view of your comments and for clarity – please see above.
